# Value contribution of trifluridine/tipiracil with bevacizumab for the treatment of metastatic colorectal cancer in Catalonia using a multicriteria decision analysis

**DOI:** 10.1080/20523211.2025.2567970

**Published:** 2025-10-13

**Authors:** Candela Calle, Elena Elez, José Luis Manzano, Maria-Estela Moreno-Martinez, Carles Pericay, Ruth Graefenhain, Asís Ariznavarreta, Luis Lizan

**Affiliations:** a Fundació Sant Francesc d’Assís, Barcelona, Spain; b Hospital Universitario Vall d’Hebrón, Barcelona, Spain; c Catalan Institute of Oncology, Hospital Germans Trials i Pujol, Barcelona, Spain; d Servei de Farmacia, Hospital de la Santa Creu i Sant Pau, Barcelona, Spain; e Hospital Universitari Mútua de Terrassa, Barcelona, Spain; f Servier España, Madrid, Spain; g Outcomes'10 a ProductLife Group Company, Castellón, Spain

**Keywords:** Colorectal cancer, colorectal neoplasms, treatment outcomes, decision making, multicriteria decision analysis, trifluridine, tipiracil, bevacizumab

## Abstract

**Background:**

Despite the alternatives available in metastatic colorectal cancer (mCRC), evaluating new therapies continues to be a challenge for clinicians and decision-makers. We aimed to generate a conceptual framework and establish the value criteria for evaluating treatments in mCRC and to apply this framework to assess the value of trifluridine/tipiracil (FTD/TPI) + bevacizumab (BEVA) in advanced lines of therapy for mCRC versus alternatives.

**Methods:**

A multicriteria decision analysis (MCDA) was conducted according to the modified EVIDEM assessment framework and the following process: defining the decision problem, selecting and structuring the related criteria identified in a literature review, weighting criteria, measurement of performance, and scoring alternatives, to evaluate of mCRC therapies in Catalonia (Spain). An expert panel of five members with experience in cancer treatment in Catalonia was involved. A hierarchical and non-hierarchical weighting of the criteria and sub-criteria was performed. An evidence matrix of available treatments was developed, and experts assigned scores comparing FTD/TPI + BEVA versus alternatives.

**Results:**

Five value criteria and 18 sub-criteria were selected to evaluate mCRC therapies. According to hierarchical method, efficacy had the highest weight, followed by safety, and lower weights for the rest: cost, guidelines/expert consensus, and epidemiology. In the efficacy dimension, overall survival (OS) had the highest weight, while severe adverse events received the highest score in terms of safety. Non-hierarchical weighting corroborated these results. Treatment with FTD/TPI + BEVA obtained a positive overall value contribution of 0.91, 0.43 points considering only comparative criteria. Comparative criteria were highly important, obtaining values contribution for efficacy, safety and cost of 0.19, 0.15 and 0.12 points, respectively. OS (0.20), progression-free survival (0.19), disease control rate (0.16) and health-related quality of life (0.16) were the most relevant sub-criteria.

**Conclusion:**

Based on this MCDA, the high value contribution of FTD/TPI + BEVA suggests its substantial benefits over the most used alternatives.

## Background

High-level health decisions are complex, mainly when they depend on the budget holder and policy decision-makers need to ensure efficient resources allocation by applying explicit and transparent procedures (Angelis & Kanavos, 2017; Angelis et al., 2017; Hsu et al., 2019; Marsh et al., 2016). Since there is controversy over whether current methodologies adequately capture the value of drugs beyond economic and efficacy criteria, various value frameworks have been developed to assist decision-makers in the new drug assessment (Angelis et al., 2020).

Using structured, formal approaches to decisions involving multiple criteria can improve decision-making quality, helping individuals or groups explore decisions that matter to them (Thokala et al., 2016). For this reason, multicriteria decision analysis (MCDA) has emerged as a complementary technique to traditional evaluation methods to support decision-makers when different medicines are available for the same indication (Gongora-Salazar et al., 2023). The use of this technique has been increasing in recent years, with particular relevance in the evaluation of new treatments for rare diseases and cancer (Campolina et al., 2022; de Andrés-Nogales et al., 2021; Lasalvia et al., 2019; Schey et al., 2020), characterised by economic and efficacy uncertainty challenges.

In recent years, several targeted therapies have been investigated and launched for treating colorectal cancer (CRC). However, despite the availability of therapeutic options, it is estimated that about 20-50% of patients will develop metastases and that 40% of these will not respond adequately to first-line treatment for metastatic CRC (mCRC) (Cervantes et al., 2023; Fernández Montes et al., 2023).

First-line treatments mainly include fluoropyrimidines, oxaliplatin, or irinotecan in combination with anti-vascular endothelial growth factor (VEGF) or anti-epidermal growth factor receptor (EGFR) therapies. When these treatments fail or are not well tolerated, it is recommended to initiate therapy with trifluridine/tipiracil (FTD/TPI) monotherapy, regorafenib, or, as recently added to guidelines, FTD/TPI combined with bevacizumab (BEVA). Other therapeutic options include targeted therapies such as retreatment of an anti-EGFR agent (cetuximab or panitumumab) or anti-EGFR with irinotecan in patients not previously treated with anti-EGFR therapies (Benson et al., 2021; Cervantes & Martinelli, 2024; Cervantes et al., 2023; Fernández Montes et al., 2023).

Given the different options, an MCDA was used to facilitate clinicians and decision-makers in evaluating new alternatives to be incorporated into the therapeutic arsenal for the treatment of mCRC. Using an MCDA approach, we aim to develop an optimal conceptual framework and establish the value criteria to be considered in the evaluation of these drugs. Subsequently, we apply this framework to assess the value of FTD/TPI + BEVA as a third line of therapy for mCRC compared to the available therapeutic alternatives.

## Methods

The MCDA was developed according to the recommendations of the International Society for Pharmacoeconomics and Outcomes Research (ISPOR) (Thokala et al., 2016). An expert panel, comprising three oncologists specialised in mCRC, one hospital pharmacist, and one healthcare manager, attended expert consultation and discussion meetings. They were selected based on their clinical experience in cancer treatment, including clinical decisions and healthcare management.

### Defining the decision problem

The aim was to gain insights into the opinions of various stakeholders on the value assessment criteria and their importance and to position FTD/TPI + BEVA in terms of relative value compared to other used treatments for mCRC in the third line in Catalonia.

As different advanced-line treatments become available, and new drugs could be included in cancer international guidelines, given the need for healthcare decision-makers to analyze the value of these technologies in a comprehensive and multi-dimensional manner, we proposed to develop a conceptual assessment framework in advanced lines for mCRC and to test it in practice with a combined treatment recently included in international guidelines, FTD/TPI + BEVA. The following drugs used in Catalonia were included in the analysis: capecitabine, regorafenib, FTD/TPI monotherapy, cetuximab, panitumumab, irinotecan monotherapy, and irinotecan + cetuximab. Even though capecitabine and irinotecan in monotherapy are not included in international clinical practice guidelines, both agents are used in Catalonia practice in patients with advanced mCRC refractory to other previous chemotherapy agents. For this reason, they have been included in the analysis.

### Selecting and structuring the related criteria

To define and establish a structured criteria matrix, the EVIDEM (Evidence and Value proposed criteria: Impact on Decision Making) (Youngkong et al., 2011) framework was used. This framework aims to evaluate medicine systematically and includes a core model containing 13 criteria adaptable to a contextual tool (Table 1).

**Table 1.** Quantitative criteria from EVIDEM framework.

Quantitative criteria	
Need for the intervention	Disease severity
	Size of the affected population
	Unmet needs
Knowledge about the intervention	Quality of evidence
	Clinical guidelines / Expert consensus
Outcomes of the intervention	Efficacy
	Safety
	Patient-reported outcomes
Type of benefit of the intervention	Type of preventive benefit
	Type of therapeutic benefit
Economic consequences of the intervention	Drug cost
	Other medical costs
	Non-medical costs

Due to the therapeutic nature of the intervention, it was considered to exclude the *type of preventive benefit* criterion from the MCDA matrix.

A literature review was also conducted (February 2024) to provide information for each of the 12 EVIDEM criteria included in the matrix. The search was performed through the PubMed search engine using specific terms of interest combined by Boolean operators (AND, OR) to screen publications from 2021 to 2024 in English or Spanish, including articles based on phase III-IV clinical trials, observational studies, reviews, or meta-analysis. The review was supplemented by a review of grey literature sources (Clinicatrials.gov, websites of health regulatory authorities such as European Medicines Agency [EMA], Agencia Española de Medicamentos y Productos Sanitarios (AEMPS) and Spanish Ministry of Health, regional health technologies organisations/agencies, and Spanish scientific societies).

The expert panel completed a questionnaire to establish the relevance of the criteria and sub-criteria included in the criteria matrix (Supplemental File S1). The questionnaire results were shared and discussed during the expert consultation meeting.

Based on the literature review, expert consultation meeting, and questionnaire results, assessment criteria and sub-criteria were set to be weighted by the participants to obtain the MCDA core model, which served as the basis for conducting the test phase.

### Weighting criteria

The expert panel weighted the criteria and sub-criteria using hierarchical and non-hierarchical methods. Both methods were used to correct potential bias.

First, the participants established a hierarchical weighting, distributing weights out of 100 points among the different criteria according to their relevance for assessing medicines in mCRC in advanced lines in Catalonia. The exercise was repeated for each sub-criteria set. This method allows ranking from the most relevant to the least relevant criteria and sub-criteria.

Second, a non-hierarchical scoring was used to establish the relevance for each criterion and sub-criterion on a Likert scale of 1-not relevant to 5-very relevant.

It was qualitatively analyzed whether similar results were obtained with both methods.

### Measurement of performance

Once the MCDA core model was set, the performance of each alternative for mCRC in advanced lines was determined using an evidence review to prepare for the test phase. A ‘performance matrix’ was developed, including all the evidence available for each criterion and sub-criterion (Supplemental File S2).

### Scoring alternatives

A second questionnaire (Supplemental File S2) was developed to collect the experts’ scores for the mCRC alternatives based on the respective criteria. FTD/TPI + BEVA was compared with the following treatments available in Catalonia: capecitabine, regorafenib, FTD/TPI as monotherapy, cetuximab, panitumumab or irinotecan as monotherapy, and irinotecan combined with cetuximab. The scoring scale was set according to the score criteria definitions to range from 0 to 5 for non-comparative criteria and sub-criteria (common to all the alternatives) and from – 5–5 for comparative criteria and sub-criteria to compare FTD/TPI + BEVA with different interventions (a score of 5 means that FTD/TPI + BEVA is much better than the comparator in the criterion analyzed, whilst a score of −5, that FTD/TPI + BEVA is much worse than the comparator and 0, that there are no differences between FTD/TPI + BEVA and the alternative drugs evaluated). The meanings of the high and low scores are described in Supplemental File S2.

### Data analysis

For each criterion/sub-criterion, the mean, standard deviation (SD), and range of minimum and maximum scores were calculated. The value contribution (Vx) of each quantitative criterion was then calculated as the product of its normalised weights (Wx, ∑ Wx = 1) based on the hierarchical method and standardised score (Sx = score/5). These results are shown on a scale of −1 and 1, where −1 means the worst impact, and 1 means the best impact. The overall value contribution was the sum of each value contribution criterion (V = ∑ Vx).

The analysis was performed using STATA version 14 (STATA Corp., LP, College Station, TX, USA).

## Results

### Selecting and structuring the related criteria

**Figure 1.**
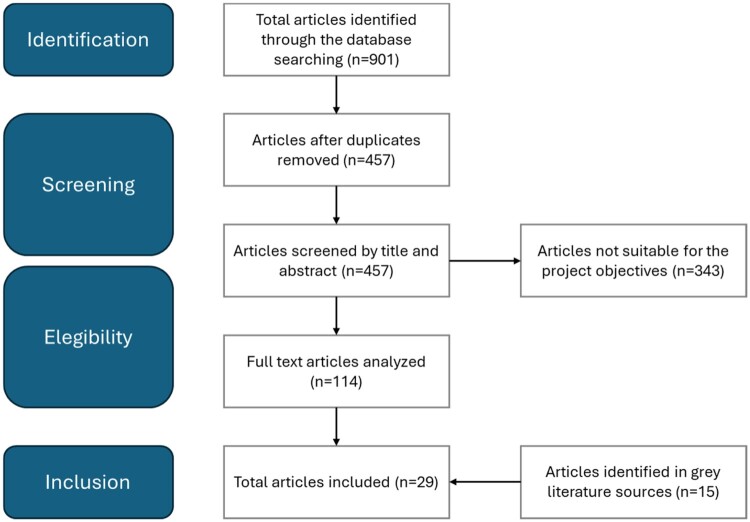
PRISMA flowchart of the literature review for identifying criteria.

Twenty-nine publications were reviewed to provide information on EVIDEM criteria (Figure 1).

The results of the expert consultation and questionnaire regarding which criteria and sub-criteria should be considered for the treatment of mCRC in advanced lines are shown in Table 2.

**Table 2.** Criteria and sub-criteria resulting from the literature review and expert consensus.

Quantitative criteria		Sub-criteria
Need for the intervention	Size of affected population – Epidemiology	Incidence and prevalence
		Percentage in relation to other cancers
Knowledge about the intervention	Clinical guidelines / Expert consensus	Guidelines/recommendations
		Real-world evidence
Outcomes of the intervention	Efficacy	Overall survival
		Progression-free survival
		Response rate
		Duration of response
		Disease control rate
		Time to progression
	Safety	Severe adverse events
		Very common and common adverse events
		Uncommon adverse events
		Impact of adverse events on health-related quality of life
	Patient-reported outcomes	*Health-related quality of life (included in efficacy)*
Economic consequences of the intervention	Cost	Drug cost
	Other medical costs	*Other treatment direct costs (included in cost)*
	Non-medical costs	*Treatment indirect costs (included in cost)*

### Weighting criteria

**Figure 2.**
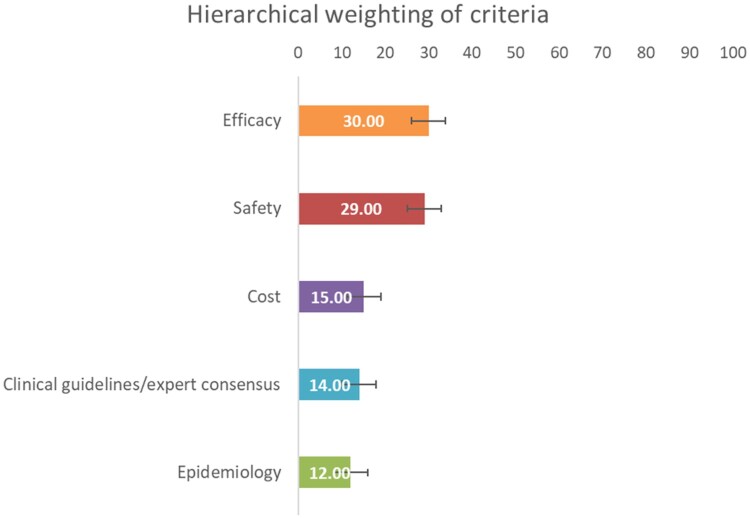
Results of the hierarchical weighting of assessment criteria.

According to the hierarchical method, *efficacy* had the highest weight, closely followed by the *safety* criteria, and with lower weights, the rest of the criteria: the *cost*, the *clinical guidelines/expert consensus,* and the *epidemiology* (Figure 2). Based on clinical guidelines, not all therapeutic alternatives showed the same level of evidence (Cervantes & Martinelli, 2024; Fernández Montes et al., 2023).

**Figure 3.**
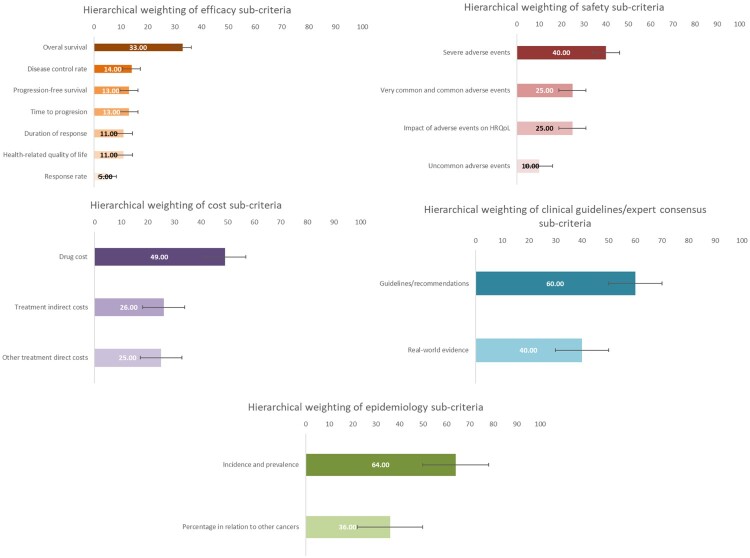
Results of the hierarchical weighting of assessment sub-criteria.

In the efficacy dimension, the *overall survival* had the highest weight, while in terms of safety, *severe adverse events* received the highest score (Figure 3). Other safety sub-criteria had relatively high weights, such as *very common and common adverse events* and *impact of adverse events on health-related quality of life (HRQoL).*

**Figure 4.**
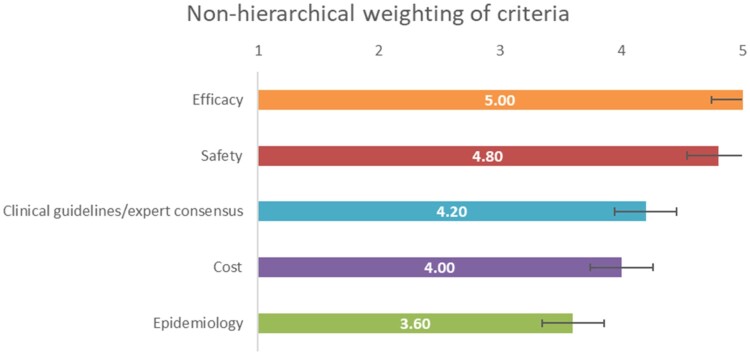
Results of the non-hierarchical weighting of assessment criteria.

The results of non-hierarchical weighting also indicate the highest scores for *efficacy* and *safety* (Figure 4). However, *cost*, *clinical guidelines/expert consensus, and epidemiology were weighted differently, the latter being the fourth criterion in terms* of weighting.

**Figure 5.**
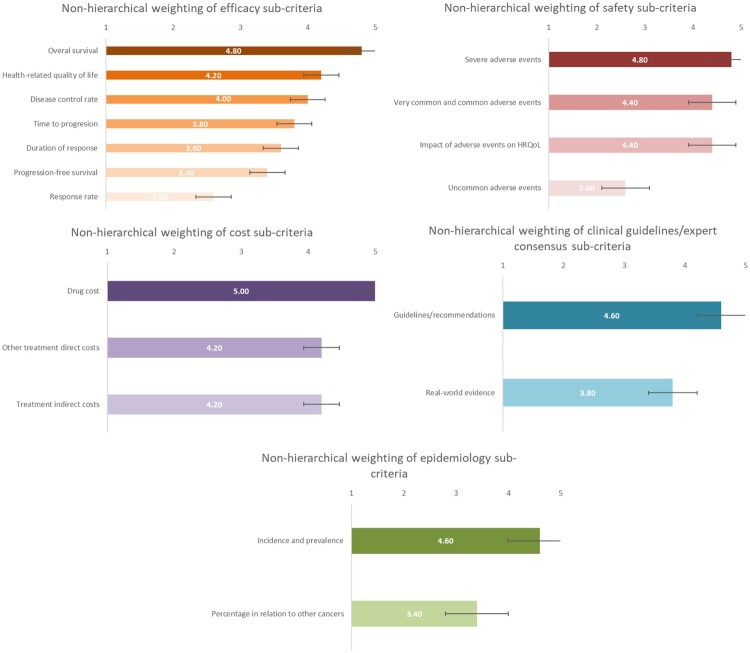
Results of the non-hierarchical weighting of assessment sub-criteria.

In line with hierarchical weighting results, the *overall survival* and *severe adverse events* reached two of the highest weights, while *drug cost* received a similar weight to *overall survival* (Figure 5).

### Measurement of performance and scoring alternatives

The performance of the criteria and sub-criteria was estimated according to their comparativeness for different alternatives for mCRC in advanced lines (Table 3).

**Table 3.** Comparative and non-comparative criteria and sub-criteria included in the evaluation of FTD/TPI + BEVA versus alternatives.

Comparative criteria	Non-comparative criteria
Efficacy	Epidemiology
Overall survival	Incidence and prevalence
Progression-free survival	Percentage in relation to other cancers
Response rate	Clinical guidelines/expert consensus
Duration of response	Guidelines/recommendations
Disease control rate	Real-world evidence
Time to progression	
Health-related quality of life	
Safety	
Severe adverse events	
Very common and common adverse events	
Uncommon adverse events	
Impact of adverse events on health-related quality of life	
Cost	
Drug cost	
Other treatment direct costs	
Treatment indirect costs	

The overall scores obtained for the treatment of mCRC in advanced lines considering comparative criteria are shown in Supplemental Figure S1 while considering both comparative and non-comparative criteria are shown in Supplemental Figure S2.

**Figure 6.**
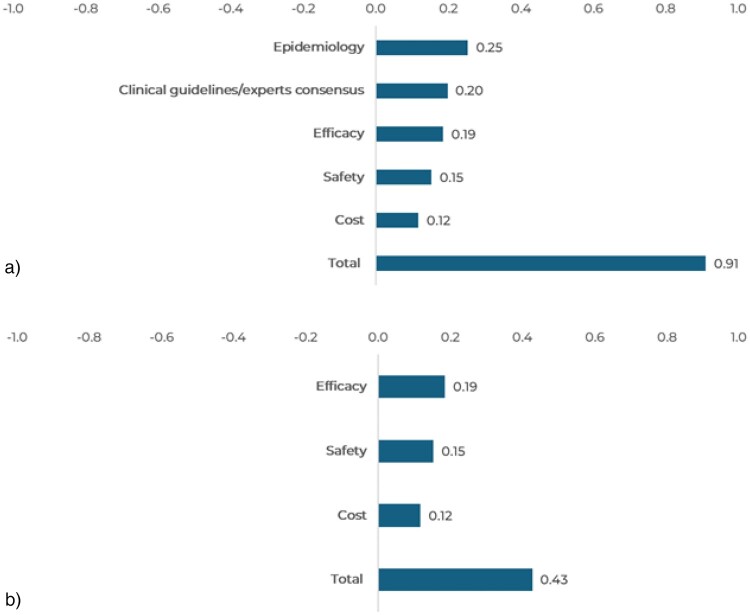
Value contribution of each criterion: FTD/TPI + BEVA vs alternatives. A. Value contribution for comparative and non-comparative criteria. B. Value contribution for comparative criteria.

Treatment with FTD/TPI + BEVA obtained a positive overall value contribution of 0.91 points (Figure 6A) compared to alternatives and 0.43 considering only comparative criteria (Figure 6B). To reach this value, comparative criteria and sub-criteria were critical, obtaining values contribution for efficacy, safety, and cost of 0.19, 0.15 and 0.12 points, respectively, compared to alternatives currently available.

**Figure 7.**
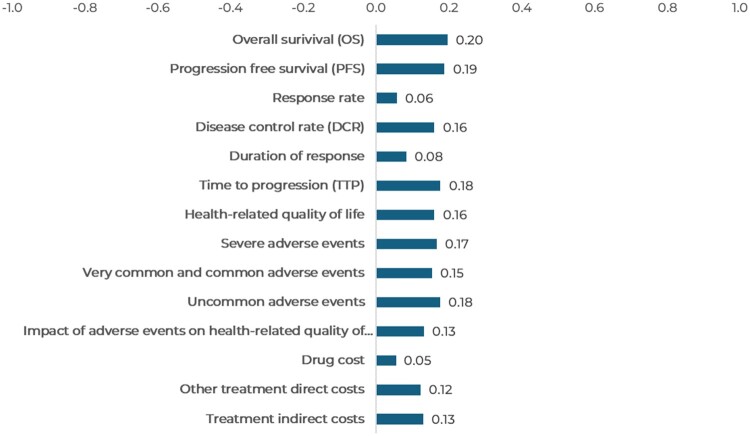
Value contribution of each sub-criteria: FTD/TPI + BEVA vs alternatives.

Analyzing efficacy in-depth, the most relevant sub-criteria were overall survival (0.20), progression-free survival (0.19), disease control rate (0.16), and health-related quality of life (0.16) based on weighting attribution (Figure 7). The safety domain also achieved positive scores in all the sub-criteria analyzed (range of 0.13-0.17 points). In terms of costs, although the pharmacological cost received the lowest, albeit positive, score (0.05), non-pharmacological medical treatment-related costs (0.12) and indirect costs (0.13) received a higher score.

## Discussion

In recent years, promising new oncological treatments have emerged; therefore, assessing treatment value has become increasingly challenging. Using an MCDA as an evaluation method allows us to simultaneously consider a wide range of criteria and capture various components of the added value of a drug (Phelps & Madhavan, 2017; Rutten-van Mölken et al., 2018).

The value framework presented here proposes a comprehensive and holistic set of criteria for mCRC treatment evaluation. Our results suggest that FTD/TPI + BEVA provides significant value for the mCRC management compared to available alternatives in Catalonia. FTD/TPI + BEVA obtained an overall score of 0.43, which indicates that it may provide more significant benefit for the domains assessed compared to other options currently available in practice in the region. The main comparative criteria contributing to the value of this treatment were *efficacy* and *safety,* whereas *cost* was the criterion that contributed the least.

Other multicriteria evaluation studies have been conducted on mCRC in different settings, with mixed results (Angelis et al., 2017; Hsu et al., 2019). Other studies have previously focused on assessing the value of available treatments in mCRC. Hsu JC et al focused on targeted therapies, analyzing the order of weights of different dimensions or evaluation criteria and ranking the value of these therapies (Hsu et al., 2019). Their results showed that the clinical dimension (including efficacy, safety, and HRQoL) had the highest weight, followed by the economic and social dimensions. In line with our results in the hierarchical weighting, the ‘comparative efficacy’ and ‘comparative safety’ included in the clinical dimension were the criteria with the highest weights, followed by the ‘cost-effectiveness’ criterion. Similarly, in the study by Angelis et al. overall survival and adverse events were the criteria with the highest relative weight compared to the rest of the criteria analyzed; however, the next criterion with the greatest relative weight was HRQoL over medical costs. In accordance with the approach of the present work, HRQoL was included as a sub-criterion of efficacy, therefore our results were similar to those found in previous studies (Angelis et al., 2017).

Traditionally, the economic value of new therapies is assessed through budget impact and cost-effectiveness analyses. These components might become less relevant in the evaluation, as more alternatives become available, less evidence is available on the comparative effectiveness, and the population susceptible to receive these treatments is reduced. Different value frameworks have been developed by recognised HTA organisations to define criteria that should be considered in a more comprehensive evaluation process (Zelei et al., 2021). Previous research has analyzed the applicability of MCDA in coverage and reimbursement decisions of innovative healthcare technologies (Goetghebeur et al., 2012; Marsh et al., 2018; Thokala et al., 2016). A recent systematic review on the use of MCDA to support decision making found that the results of 40% of the included studies were useful for informing health authorities and HTA agencies in making decisions about drug funding (Gongora-Salazar et al., 2023). In addition, this review also indicated that half of the articles analyzed included cost as one of the attributes to be analyzed.

According to other authors, the MCDA should be seen as a more complete way of measuring the benefit of a therapy, being a complement to traditional analysis rather than a substitute for it. Each region should include the MCDA methodology as a systematic procedure to complement the process of evaluation of the added clinical value for innovative medicines when different treatment options are available, and decision-making becomes more complex. This methodology makes it possible to incorporate decision-maker perspectives in a well-structured and transparent methodology, simplifying and standardising the value-based decision-making process (Blythe et al., 2019). In addition, the systematic evaluation of different evidence sources enabled the identification of strengths and weaknesses for each drug. It could influence both the clinical development process and its use in clinical practice (Angelis et al., 2017; Hsu et al., 2019).

This study has some limitations. Firstly, the expert panel consisted of a small sample of five experts; selected for their clinical and decision-making experience in cancer management, as well as opinion leaders assessing the therapeutic and economic value of new drugs. Even though the sample size can be considered small, it was representative of the phenomenon under investigation, given the focused nature of the study, and the structured and extremely detailed methodology. Previous studies have shown how the use of this methodology, due to its capacity to deeply and broadly analyze complex problems with conflicting criteria, can be limited to small samples of participants, composed of experts previously involved in the resolution of these problems (Álvarez-Román et al., 2019; Badia et al., 2024; Villarubia et al., 2023). Secondly, our study focused on Catalonia context, which may differ from other regions with different available therapeutic options. However, due to the complex and multidimensional nature of the drug evaluation, the importance of this study resides in the methodology used, which provides a structured and transparent approach, allowing for a more comprehensive process for decision-makers. This could explain why our results resemble the results obtained in previous studies, indicating that these findings might be extrapolable to other settings. Finally, this study, like current HTA processes, fails to incorporate the patient’s perspective. According to previous studies, the perspective of patients and healthcare professionals should be routinely incorporated into assessments (Garrett et al., 2022; Zimmermann et al., 2023). However, there is no standardised approach for incorporating their perspective into HTA, although other authors have explored how MCDA could be a suitable tool (Badia et al., 2019). Nevertheless, there are certain challenges to be resolved, such as raising awareness among health authorities of the importance of integrating patients as agents in evaluation committees, or the need for ‘expert’ patients who are able to understand and assess the different evaluation criteria (Badia et al., 2019). Future studies could focus on exploring the impact of patient preferences on assessment and resolving how their perspective can be fully and routinely considered.

## Conclusion

Drug value assessment should consider multiple dimensions and criteria. The value framework presented will serve as a basis for future evaluations of mCRC in advanced lines of treatment in Catalonia. It may encourage a better understanding and analysis of the value provided by a novel therapeutic intervention. Based on this MCDA, the high-value contribution of FTD/TPI + BEVA suggests its substantial benefits over the most used alternatives. The scores it reaches are close to the maximum possible value, indicating the importance of the disease treated and the benefits of the treatment. This tool can bring greater objectivity to evaluating new drugs from the cost to the patient's perspective.

## Supplementary Material

Supplemental File S2

Supplemental File S1

## Data Availability

The datasets used and/or analyzed during the current study are available from the corresponding author upon reasonable request. All data generated or analyzed during this study are included in this published article [and its supplementary information files].

## Abbreviations

**Abbreviations:** AEMPS: Agencia Española de Medicamentos y Productos Sanitarios; BEVA: bevacizumab; CRC: colorectal cancer; EGFR: anti-epidermal growth factor receptor; EMA: European Medicines Agency; EVIDEM: evidence and value: impact on decision-making; FTD/TPI: trifluridine/tipiracil; HRQoL: health-related quality of life; ISPOR: international society for pharmacoeconomics and outcomes research; MCDA: multicriteria decision analysis; mCRC: metastatic colorectal cancer; SD: standard derivation; Sx: standardised score; VEGF: anti-vascular endothelial growth factor; Vx: value contribution
