# Supplementary material for: Value contribution of trifluridine/tipiracil with bevacizumab for the treatment of metastatic colorectal cancer in Catalonia using a multicriteria decision analysis
Source: J Pharm Policy Pract. 2025 Oct 13;18(1):2567970. doi: 10.1080/20523211.2025.2567970 (PMC12710260; doi:10.1080/20523211.2025.2567970)
Supplement: Supplemental File S2 [file JPPP_A_2567970_SM8810.docx]

**Supplementary file 2 – Matrix and Questionnaire 2**

Tabla de contenido

Introducción2

Puntuación de los criterios y subcriterios3

1. Criterios no comparativos3
2. Criterios comparativos5

Referencias13

**Introducción**

El cáncer colorrectal (CCR) es la tercera entidad tumoral más frecuente en todo el mundo, con 1,1 millones de nuevos casos al año (A. Cervantes et al., 2023; Pretzsch et al., 2019). En España, el CCR es el tumor más frecuentemente diagnosticado en 2022, según datos proporcionados por la Sociedad Española de Oncología Médica (SEOM) (Gaceta Medica, 2022).

Se estima que aproximadamente entre el 20 y el 50% de los pacientes con enfermedad inicialmente localizada desarrollarán metástasis. La localización más frecuente de la metástasis es el hígado, después el pulmón, el peritoneo y los ganglios linfáticos (A. Cervantes et al., 2023).

Respecto a su tratamiento, las opciones terapéuticas se basan en el estadio de la enfermedad según la clasificación TNM (Benson et al., 2021; A. e. a. Cervantes, 2023; Fernandez Montes et al., 2023). Para la mayoría de los pacientes en estadio I y II (sin propagación a los ganglios linfáticos adyacentes, ni a sitios distantes) el tratamiento se basa en la cirugía, y para los pacientes en estadio III (sin propagación a sitios distantes) la cirugía y la quimioterapia adyuvante es el tratamiento estándar (Carrertero Colomer, 2005). En los pacientes con estadio IV o metastásico (CCRm), no se dispone de un tratamiento curativo, y el objetivo del tratamiento actual es prolongar la supervivencia global (SG) o libre de progresión (SLP) y mejorar la calidad de vida del paciente (AEMPS, 2016b).

En función de la resecabilidad del tumor primario y de las metástasis identificadas se dispone de diferentes abordajes terapéuticos (AEMPS, 2016b, 2016c). Cerca del 40% de los pacientes con CCRm no responden adecuadamente a las primeras líneas de tratamiento, y se requiere una tercera línea o sucesivas (Fernandez Montes et al., 2023). Cuando el CCRm ha progresado tras dos líneas de tratamiento previas, que incluyen principalmente fluoropirimidinas, platinos o irinotecán en combinación con anti-factor de crecimiento endotelial vascular (VEGF), o anti-receptor del factor de crecimiento epidérmico (EGFRs), las terapias en tercera línea han demostrado mejorar la supervivencia global y libre de progresión. Principalmente, se recomienda iniciar terapia con trifluridina-tipiracilo (FTD/TPI) en monoterapia, FTD/TPI con bevacizumab, o regorafenib (Benson et al., 2021; A. Cervantes et al., 2023; A. e. a. Cervantes, 2023; Fernandez Montes et al., 2023). Otras opciones terapéuticas, incluyen el uso de terapias dirigidas con retratamiento de un anti-EGFR (cetuximab o panitumumab), o anti-EGFR con irinotecán en pacientes no tratados previamente con anti-EGFR (A. Cervantes et al., 2023; Fernandez Montes et al., 2023).

El valor añadido que una nueva terapia aporta al manejo del CCRm debe basarse en la evidencia científica disponible, y tener en cuenta criterios de valoración que vayan más allá del precio del fármaco. Para facilitar a clínicos y decisores sanitarios la valoración de nuevas alternativas en CCRm en líneas avanzadas, se llevó a cabo un análisis de decisión multicriterio (MCDA, de sus siglas en inglés *multicriteria decision analysis* en Cataluña, con el objetivo de crear un marco conceptual óptimo, estableciendo cuáles son los criterios a considerar en la evaluación de estos medicamentos en la región.

En base a los criterios y subcriterios de valor consensuados en el MCDA, se ha desarrollado la siguiente matriz que recoge la evidencia disponible de la alternativa terapéutica FTD/TPI con bevacizumab frente a los fármacos utilizados según práctica clínica en CCRm en líneas avanzadas: **capecitabina, regorafenib, trifluridina/tipiracilo (FTD/TPI), cetuximab, panitumumab o irinotecán en monoterapia, e irinotecán combinado con cetuximab.**

Se le invita a que asigne a cada criterio y subcriterio, la puntuación que considere de acuerdo con la evidencia disponible descrita en la matriz. En caso de no disponer de información, deberá asignar la puntuación que considere oportuna de acuerdo con su experiencia y opinión.

La escala de puntuación utilizada es:

- Para los criterios no comparativos (comunes a cualquiera de las alternativas):
- Epidemiología: indicará de 0 a + 5, según si la enfermedad no es nada prevalente (0) hasta muy prevalente (5), para el criterio y cada subcriterio descritos.
- Guías de práctica clínica/consenso de expertos: indicará de 0 a + 5, según si la enfermedad y el tratamiento no están nada definidos (0) hasta muy definidos (5), para el criterio y cada subcriterio descritos.
- Para los criterios comparativos:
  - Eficacia: indicará de -5 a + 5, según si considera que FTD/TPI con bevacizumab es la peor opción terapéutica frente al resto (-5), que no hay diferencias con las alternativas a comparar (0), o FTD/TPI con bevacizumab es la mejor opción (5).
  - Seguridad: indicará de -5 a + 5, según si considera que FTD/TPI con bevacizumab es la opción terapéutica menos segura y tolerable (-5), que no hay diferencias con las alternativas a comparar (0), o FTD/TPI con bevacizumab es la más segura y tolerable (5).
  - Coste: indicará de -5 a + 5, según si considera que FTD/TPI con bevacizumab es la peor opción terapéutica frente al resto (-5), que no hay diferencias con las alternativas a comparar (0), o FTD/TPI con bevacizumab es la mejor opción (5).

Los resultados de la puntuación serán presentados al comité científico en la próxima reunión online del **27 de mayo** a las 16:00h.

Tipo de perfil profesional (indique solo UNA opción, la que considere que más aplica a su perfil profesional):

- - Médico
  - Farmacéutico Hospitalario
  - Decisor en salud
  - Otro (especifique): ………………………………

A continuación, lea detenidamente el contenido que se presenta en la columna central con la evidencia disponible y otorgue en las filas inferiores de cada matriz las puntaciones que para cada criterio y subcriterio considere oportunas. Tenga en cuenta que esta matriz simplemente es un instrumento de ayuda a sus reflexiones y no pretende ningún otro objetivo añadido. Tras la puntuación de cada criterio, añada la reflexión sobre su puntuación en el espacio dedicado para ello si lo considera necesario.

Muchas gracias por su participación

# **Puntuación de los criterios y subcriterios**

## **Criterios no comparativos**

| CRITERIO | EVIDENCIA DISPONIBLE | | | | | |
| --- | --- | --- | --- | --- | --- | --- |
| Epidemiología | | | | | | |
| Subcriterio: Incidencia y prevalencia | Según los datos de la Red Española de Registros de Cáncer (Redecan), se estiman 42.721 casos incidentes en España para el 2023(Redecan, 2023). No se han encontrado datos específicos de incidencia para el cáncer colorrectal metastásico (CCRm) en España, pero se conoce que aproximadamente entre el 15% y el 30% de los pacientes con CCR presentan metástasis al diagnóstico, y entre el 20% y el 50% de los pacientes con enfermedad inicialmente localizada desarrollarán metástasis (A. Cervantes et al., 2023). Según el documento de la Sociedad Española de Oncología Médica (SEOM) de 2024 se ha estimado que la prevalencia a los cinco años del diagnóstico de CCR a nivel mundial para el año 2020 es de 5.253.335.  En España, se estimaron unos 43.370 casos para el 2022 (Fernandez Montes et al., 2023). Teniendo en cuenta que un 50% de los pacientes progresan a metástasis, correspondería a 21.685 pacientes con CCRm(Fernandez Montes et al., 2023), de los cuales el 85% pasaran a primera línea (18.432 pacientes). Para líneas avanzadas se estiman 11.981 y 4.193 pacientes en segunda y tercera línea, respectivamente (65% y 35% de los pacientes que pasan de una línea a la siguiente) (Fernandez Montes et al., 2023; Servei Català de la Salut, 2018). | | | | | |
| Puntúe el subcriterio según considere: | 5 Muy prevalente | 4 | 3 | 2 | 1 | 0 Nada prevalente |
| Subcriterio: Porcentaje respecto al resto de cánceres: | Según la OMS, el cáncer colorrectal supone casi el 20% de los 6 canceres más comunes (cáncer de mama, de pulmón, colorrectal, de próstata, de piel y gástrico) en 2020. | | | | | |
| Puntúe el subcriterio según considere: | 5 Muy prevalente | 4 | 3 | 2 | 1 | 0 Nada prevalente |
| Puntúe el criterio Epidemiología en global | 5 Muy prevalente | 4 | 3 | 2 | 1 | 0 Nada prevalente |
| A continuación, si lo desea puede indicar el razonamiento de su ponderación: | | | | | | |

| CRITERIO | EVIDENCIA DISPONIBLE | | | | | |
| --- | --- | --- | --- | --- | --- | --- |
| Guía de práctica clínica/consenso de expertos | | | | | | |
| Subcriterio: Guías y recomendaciones | Se han identificado dos guías a nivel internacional (NCCN y ESMO), una guía española en CCR y un consenso de expertos de manejo del paciente anciano con CCR, ambos del GEMCAD, TTD y la SEOM (Benson et al., 2021; A. e. a. Cervantes, 2023; Fernandez Montes et al., 2023; Soler-Gonzalez et al., 2024). A nivel de Cataluña, se suelen seguir las guías del ICO, las ultimas guías fueron publicadas en el 2020.  Las guías ESMO, y SEOM-GEMCAD-TTD recomiendan para los pacientes con CCRm en líneas avanzadas (A. Cervantes et al., 2023; Fernandez Montes et al., 2023):   \| Opción terapéutica \| Nivel evidencia \| \| \| --- \| --- \| --- \| \| ESMO (2022-23) \| SEOM-GEMCAD-TTD (2023) \| \| FTD/TPI + bevacizumab \| IA \| IA \| \| FTD/TPI \| IA \| - \| \| Cetuximab en monoterapia \| IA* \| IIIC** \| \| Cetuximab + irinotecán \| IIB \| - \| \| Irinotecán en monoterapia \| - \| - \| \| Panitumumab en monoterapia \| IA* \| IIIC** \| \| Capecitabina en monoterapia \| - \| - \| \| Regorafenib \| IA \| IA \| \| *sólo RAS-wt y BRAF-wt en retratamiento; **en retratamiento con un anti-EGFR  Cabe destacar que, FTD/TPI con bevacizumab cuenta con la indicación a los pacientes con CCRm en líneas avanzadas independientemente de si tiene alguna mutación o no, eliminando la necesidad de realizar la secuenciación. \| \| \| | | | | | |
| Puntúe el subcriterio según considere: | 5 Muy definido el problema y el tratamiento | 4 | 3 | 2 | 1 | 0 Nada definido el problema y el tratamiento |
| Subcriterio: RWE | Las guías de práctica clínica no incluyen recomendaciones basadas en datos de vida real en tercera línea de tratamiento. Sin embargo, se dispone de datos en vida real sobre la efectividad para las opciones terapéuticas FTD/TPI + bevacizumab e Irinotecán + cetuximab:   \| **Tratamiento** \| **SG**  **mediana (95% IC)** \| **SLP**  **mediana (95% IC)** \| **TR**  **% (95% IC)** \| **DCR**  **% (95% IC)** \| \| --- \| --- \| --- \| --- \| --- \| \| Irinotecán + Cetuximab (Gathirua-Mwangi, Yang, Khan, Wu, & Afable, 2022) \| 12,0 (ND) \| 3,3 (ND) \| ND \| ND \| \| Irinotecán + Cetuximab (Maria Ignez Braghiroli et al., 2022) \| 8,9 (ND) \| ND \| 23,5 (ND) \| 50,3 (ND) \| \|  \| **Media (95% IC)** \| **Media (95% IC)** \| **TR % (95% IC)** \| **DCR % (95% IC)** \| \| FTD/TPI + BEV (Voutsadakis, 2023) (metaanálisis) \| 11,2  (10,2 – 12,2) \| 4,6  (3,6-5,6) \| 2,71 (1,1-4,3) \| 59,6 (52,1-67,2) \|   La opción terapéutica FTD/TPI + BEV presenta resultados similares en práctica clínica habitual a los alcanzados en los EECC. | | | | | |
| Puntúe el subcriterio según considere: | 5 Aportan mucha información de interés | 4 | 3 | 2 | 1 | 0 No aportan nada de información de interés |
| Puntúe el criterio Guía de práctica clínica/consenso de expertos en global | 5 Muy definido el problema y el tratamiento | 4 | 3 | 2 | 1 | 0 Nada definido el problema y el tratamiento |
| A continuación, si lo desea puede indicar el razonamiento de su ponderación: | | | | | | |

1. **Criterios comparativos**

| CRITERIO | EVIDENCIA DISPONIBLE | | | | | | | | | | |
| --- | --- | --- | --- | --- | --- | --- | --- | --- | --- | --- | --- |
| Eficacia de los tratamientos disponibles | | | | | | | | | | | |
| Subcriterios:   - Supervivencia global (SG) - Supervivencia libre de progresión (SLP) - Tasa de respuesta (TR) - Tasa de control de la enfermedad (DCR) - Duración de respuesta - Tiempo transcurrido hasta deterioro (TTP) - CVRS. - Regorafenib | En la siguiente tabla se sintetizan los resultados de eficacia de diferentes ECs:   \| **Tratamiento** \| **SG**  **mediana (95% IC)** \| **SLP**  **mediana (95% IC)** \| **TR % (95% IC)** \| **DCR % (95% IC)** \| **TTP**  **Mediana (95% IC)** \| **Duración respuesta**  **Mediana (95% IC)** \| \| --- \| --- \| --- \| --- \| --- \| --- \| --- \| \| Capecitabina (Gubanski et al, 2005) \| 6,1 (ND) \| ND \| 0 (0-13,9) \| ND \| 2,8 (ND) \| ND \| \| Irinotecán (Hartmann et al, 2004) \| 7,9 (6,1-11,1) \| ND \| 13,3 (5,1-26,8) \| 51,1 (35,8-66,3) \| 3,0 (2,0-4-1) \| 4,2 (3,2-6,0) \| \| Irinotecán + Cetuximab (Cunningham et al, 2004; BOND) \| 8,6 (ND) \| 4,1 (ND) \| 22,9 (17,5-29,1) \| 55,5 (48,6-62,2) \| ND \| 5,7 \| \| Cetuximab (Cunningham et al, 2004; BOND) \| 6,9 (ND) \| 1,5 (ND) \| 10,8 (5,7-18,1) \| 32,4 (23,9-42,0) \| ND \| 4,2 \| \| Cetuximab (Jonker et al, 2007) \| 6,1 (ND) \| 2,0 (ND) \| ND \| ND \| ND \| ND \| \| Panitumumab (Van Cutsem et al, 2007) \| 6,5 (ND) \| 2,0 (ND) \| 10 \| ND \| ND \| ND \| \| Regorafenib (Grothey et al, 2013) \| 6,4 (3,6-11,8) \| 1,9 (1,6-3,9) \| 1,0 (ND) \| 41,0 \| ND \| 2,0 (1,7-4,0) \| \| FTD/TPI (Prager et al, 2023; SUNLIGHT) \| 7,5 (6,3-8,6) \| 2,4 (2,1-3,2) \| 1,2 (0,3-3,5) \| 41,9 (ND) \| 6,3 (5,6-7,2) \| ND \| \| FTD/TPI + BEV (Prager et al, 2023; SUNLIGHT) \| 10,8  (9,4–11,8) \| 5,6  (4.5- 5,9) \| 6,1 (3,5-9,9) \| 69,5 (ND) \| 9,3 (8,3-10,6) \| ND \|  - CVRS   Debido a la toxicidad de los tratamientos para el CCRm en tercera línea, la CVRS de los pacientes se puede ver comprometida. Se dispone de escasa evidencia de las terapias en tercera línea en cuanto a la mejora en la CVRS. Los datos disponibles extraídos de los EECC y estudios adicionales se muestran en la siguiente tabla:   \| **Tratamiento** \| **Instrumento medida (n)** \| **Momento de recogida de datos (n)** \| **Resultados** \| \| --- \| --- \| --- \| --- \| \| Cetuximab (Price et al, 2014; ASPECCT) \| EuroQOL EQ-5D \| De inicio hasta fin de tratamiento o progresión hasta semana 85  (150) \| Escala visual analógica (EVA)*:  4,0 (0,9-7,1) \| \| Panitumumab (Price et al, 2014; ASPECCT) \| EuroQOL EQ-5D \| Desde inicio hasta fin de tratamiento o progresión hasta semana 85  (143) \| EVA*:  2,3 (-0,9-5,5) \| \| Regorafenib (Grothey et al, 2013) \| EuroQOL EQ-5D \| Desde inicio hasta fin de tratamiento \| Cambio desde media de 65,4 (DE 19,6) puntos en la EVA*, al inicio del tratamiento, hasta 55,5 puntos al final del tratamiento. \| \| FTD/TPI (Prager et al, 2023; SUNLIGHT) \| EORTC-QLQ-C30 \| En cada ciclo  (480) \| Mediana de tiempo hasta deterioro de la salud global (GHS): 4,7 meses \| \| FTD/TPI + BEV (Prager et al, 2023; SUNLIGHT) \| EORTC-QLQ-C30 \| En cada ciclo  (480) \| Mediana de tiempo hasta deterioro de la salud global (GHS): 8,5 meses \|   *En la EVA se pide a los encuestados que valoren su estado de salud actual en una escala de 0 a 100, en la que 0 es el «Peor estado de salud imaginable» y 100 el «Mejor estado de salud imaginable». La puntuación de la EVA se determina observando el punto en el que la línea trazada a mano por el participante se cruza con la escala. | | | | | | | | | | |
| Puntúe los siguientes subcriterios según considere: | | | | | | | | | | | |
| Supervivencia global (SG) | ☐ 5 FTD/TPI + bevacizumab es la mejor opción | ☐ 4 | ☐ 3 | ☐ 2 | ☐ 1 | ☐ 0 | ☐ -1 | ☐ -2 | ☐ -3 | ☐ -4 | ☐ -5 FTD/TPI + bevacizumab es la peor opción |
| Supervivencia libre de progresión (SLP) | ☐ 5 FTD/TPI + bevacizumab es la mejor opción | ☐ 4 | ☐ 3 | ☐ 2 | ☐ 1 | ☐ 0 | ☐ -1 | ☐ -2 | ☐ -3 | ☐ -4 | ☐ -5 FTD/TPI + bevacizumab es la peor opción |
| Tasa de respuesta (TR) | ☐ 5 FTD/TPI + bevacizumab es la mejor opción | ☐ 4 | ☐ 3 | ☐ 2 | ☐ 1 | ☐ 0 | ☐ -1 | ☐ -2 | ☐ -3 | ☐ -4 | ☐ -5 FTD/TPI + bevacizumab es la peor opción |
| Tasa de control de la enfermedad (DCR) | ☐ 5 FTD/TPI + bevacizumab es la mejor opción | ☐ 4 | ☐ 3 | ☐ 2 | ☐ 1 | ☐ 0 | ☐ -1 | ☐ -2 | ☐ -3 | ☐ -4 | ☐ -5 FTD/TPI + bevacizumab es la peor opción |
| Duración de respuesta | ☐ 5 FTD/TPI + bevacizumab es la mejor opción | ☐ 4 | ☐ 3 | ☐ 2 | ☐ 1 | ☐ 0 | ☐ -1 | ☐ -2 | ☐ -3 | ☐ -4 | ☐ -5 FTD/TPI + bevacizumab es la peor opción |
| Tiempo transcurrido hasta deterioro (TTP) | ☐ 5 FTD/TPI + bevacizumab es la mejor opción | ☐ 4 | ☐ 3 | ☐ 2 | ☐ 1 | ☐ 0 | ☐ -1 | ☐ -2 | ☐ -3 | ☐ -4 | ☐ -5 FTD/TPI + bevacizumab es la peor opción |
| CVRS | ☐ 5 FTD/TPI + bevacizumab es la mejor opción | ☐ 4 | ☐ 3 | ☐ 2 | ☐ 1 | ☐ 0 | ☐ -1 | ☐ -2 | ☐ -3 | ☐ -4 | ☐ -5 FTD/TPI + bevacizumab es la peor opción |
| Puntúe el criterio Eficacia en global | ☐ 5 FTD/TPI + bevacizumab es la mejor opción | ☐ 4 | ☐ 3 | ☐ 2 | ☐ 1 | ☐ 0 | ☐ -1 | ☐ -2 | ☐ -3 | ☐ -4 | ☐ -5 FTD/TPI + bevacizumab es la peor opción |
| A continuación, si lo desea puede indicar el razonamiento de su ponderación: | | | | | | | | | | | |

| CRITERIO | EVIDENCIA DISPONIBLE | | | | | | | | | | |
| --- | --- | --- | --- | --- | --- | --- | --- | --- | --- | --- | --- |
| Seguridad | | | | | | | | | | | |
| Subcriterios:   - Eventos adversos graves - Eventos adversos frecuentes y muy frecuentes - Eventos adversos poco frecuentes - Afectación de los eventos adversos en la CVRS | - Eventos adversos graves:   Entre las distintas alternativas terapéuticas del CCRm en líneas avanzadas los EA graves (grados III/IV) encontrados en los EECC fueron (no se incluyó Capecitabina por no encontrarse descritos en el ensayo de Gubanski et al, 2005):   \| **Eventos adversos graves** \| **Irinotecán (Hartmann et al, 2004)**  **N (%)** \| **Irinotecán +Cetuximab (Cunningham et al, 2004; BOND) N (%)** \| **Cetuximab (Cunningham et al, 2004; BOND; Jonker et al, 2007)**  **N (%)** \| **Panitumumab (Van Cutsem et al, 2007)**  **N (%)** \| **Regorafenib** \| **FTD/TPI + BEV (Prager et al, 2023; SUNLIGHT)**  **N (%)** \| \| --- \| --- \| --- \| --- \| --- \| --- \| --- \| \| Neutropenia \| 7 (14,0) \| 20 (9,4) \| 0 (0) \| - \| - \| 106 (43.1) \| \| Nausea \| 2 (4,0) \| - \| 16 (5,6) \| 2 (1,0) \| 4 (1,0) \| 4 (1.6) \| \| Anemia \| 3 (6,0) \| 10 (4,7) \| 3 (2,6) \| - \| 14 (2,8) \| 15 (6.1) \| \| Astenia \| 1 (2,0) \| 29 (13,7) \| 12 (10,4) \| 7 (3,1) \| - \| 10 (4.1) \| \| Fatiga \| - \| - \| 95 (33,0) \| 10 (4,0) \| 48 (9,6) \| 3 (1.2) \| \| Diarrea \| 12 (24,0) \| 45 (21,2) \| 2 (1,7) \| 3 (1,0) \| 36 (7,2) \| 2 (0.8) \| \| Disminución de apetito \| - \| - \| 24 (8,3) \| 8 (3,5) \| 16 (3,2) \| 2 (0.8) \| \| Vómitos \| 4 (8,0) \| - \| 16 (5,6) \| 5 (2,0) \| 3 (0,6) \| 2 (0.8) \| \| Reacciones cutáneas \| - \|  \| 34 (11,8) \| 24 (10,5) \| 112 (24,4) \| - \| \| Erupción similar a acné \| - \| 20 (9,4) \| 6 (5,2) \| 17 (7,0) \| - \| - \| \| Disnea \| - \| 3 (1,4) \| 47 (16,3) (Jonker 2007)  15 (13,0) (Cunningham 2004) \| 11 (4,8) \| 1 \| - \| \| Disminución del número de neutrófilos \| - \| - \| - \| - \| - \| 22 (8.9) \| \| Hipertensión \| - \| - \| - \| - \| 36 (7,0) \| 14 (5.7) \| \| Infección no neutropénica \| - \| - \| 37 (12,8) \| - \| - \| - \| \| Dolor abdominal \| - \| 7 (3,3) \| 38 (13,2) (Jonker 2007)  6 (5,2) (Cunningham 2004) \| - \| 1 (0,2) \| - \| \| Estreñimiento \| 2 (4,0) \| - \| 10 (3,5) \| 6 (3,0) \| 0 (0,0) \| - \| \| Dolor, dolor de espalda \| 7 (14,0) \|  \|  \|  \| 2 (0,4) \|  \| \| Aumento Bilirrubina \| 8 (16,0) \| - \| - \| 4 (2,0) \| 10 (2,0) \| - \|  - Eventos adversos muy frecuentes y frecuentes   Se consideran EA muy frecuentes aquellos eventos que pueden afectar a más de 1 de cada 10 personas y EA frecuentes; aquellos eventos que pueden afectar hasta 1 de cada 100 personas.   \| Eventos adversos muy frecuentes \| \| \| \| \| \| \| \| --- \| --- \| --- \| --- \| --- \| --- \| --- \| \| Capecitabina \| Irinotecán \| Cetuximab \| Irinotecán +Cetuximab \| Panitumumab \| Regorafenib \| FTD/TPI + BEV \| \| Síndrome eritrodisestesia palmo-plantar, diarrea, Vómitos, Náuseas, Estomatitis, Dolor abdominal, fatiga, Astenia, Anorexia \| Neutropenia, Anemia, Diarrea tardía, Vómitos, Náuseas, Dolor abdominal, Alopecia, Inflamación de mucosas, Pirexia, Astenia \| Hipomagnesemia, Aumento en los niveles de enzimas hepáticas, Reacciones cutáneas, Mucositis \| Las esperadas de ambos tratamientos. Sin evidencia de más complicaciones por combinación. \| Conjuntivitis, Paroniquia, Anemia, Hipopotasemia, Hipomagnesemia  Apetito disminuido, Insomnio, Disnea, Tos, Diarrea, Náuseas, Vómitos  Dolor abdominal  Estomatitis  Estreñimiento  Reacciones cutáneas, Dolor de espalda, Fatiga, Pirexia, Astenia, Inflamación de la mucosa, Edema periférico \| Infección, Trombocitopenia, Anemia, Disminución del apetito y de la ingesta de alimentos, Cefalea, Hemorragia, Hipertensión, Disfonía, Diarrea, Estomatitis, Vómitos, Náuseas, Hiperbilirrubinemia, Reacción cutánea mano-pie, Exantema, Alopecia, Astenia, fatiga, Dolor, Fiebre, Mucositis, Pérdida de peso \| Neutropenia, Anemia, Trombocitopenia, Apetito disminuido, Diarrea, Vómitos, Náuseas, Estomatitis, Fatiga \|  \| Eventos adversos frecuentes \| \| \| \| \| \| \| \| --- \| --- \| --- \| --- \| --- \| --- \| --- \| \| Capecitabina \| Irinotecán \| Cetuximab \| Irinotecán +Cetuximab \| Panitumumab \| Regorafenib \| FTD/TPI + BEV \| \| Rash, alopecia, eritema, sequedad de la piel, prurito, hiperpigmentación de la piel, dermatitis, rash macular; descamación cutánea, alteración de la pigmentación, alteración ungueal, Estreñimiento, dolor del tracto superior del abdomen, dispepsia, flatulencia, sequedad de boca, incontinencia fecal, hemorragia gastrointestinal, Pirexia, letargia, edema, periférico, malestar, dolor torácico no cardíaco, Deshidratación, disminución del apetito, Cefalea, letargia, vértigo parestesia, disgeusia, Aumento del lagrimeo, conjuntivitis, irritación ocular, Hiperbilirrubinemia/bilirrubina sanguínea/aumento de la bilirrubina sanguínea, Disnea, epistaxis, tos, rinorrea, Dolor en las extremidades, dolor de espalda, artralgia, Disminución de peso, alteraciones en las pruebas de la función hepática, Neutropenia, anemia, Insomnio, depresión, Herpes simple, nasofaringitis, infecciones del tracto, respiratorio inferior, Tromboflebitis \| Infección, Trombocitopenia, Neutropenia febril, Estreñimiento, Aumento: Creatinina, transaminasas, bilirrubina y fosfatasa alcalina en sangre \| Deshidratación, especialmente secundaria a diarrea o mucositis; hipocalcemia, Anorexia, Cefalea  Conjuntivitis, Diarrea, náuseas; vómitos, Reacciones graves relacionadas con la perfusión \| Las esperadas de ambos tratamientos. Sin evidencia de más complicaciones por combinación. \| Erupción pustulosa, Celulitis,  Infección del tracto urinario, Foliculitis, Infección localizada, Hipersensibilidad, Hipocalcemia, deshidratación , Hiperglucemia, Hipofosfatemia, Ansiedad, Cefalea, Mareo, Blefaritis, Crecimiento pestañas, Lagrimeo aumentado, Hiperemia ocular, Xeroftalmía, Prurito ocular, Irritación del ojo, Taquicardia, Trombosis venosa profunda, Hipotensión, Hipertensión, Rubor, Embolia pulmonar, Epistaxis, Enfermedad por reflujo gastroesofágico, Úlcera cutánea, Exfoliación de la piel, Erupción exfoliativa, Dermatitis, Erupción: papular, pruriginosa, eritematosa, generalizada, macular, maculo-papular, Lesión de la piel, Toxicidad cutánea, Costra, Hipertricosis, Onicoclasia, Alteraciones de las uñas, Hiperhidrosis, S. eritrodisestesia palmo-plantar, Dolor extremidades, Molestias en el pecho, Dolor, Escalofríos, Disminución magnesio en sangre. \| Leucopenia, Hipotiroidismo, Hipopotasemia, Hipofosfatemia, Hipocalcemia, Hiponatremia, Hipomagnesemia, Hiperuricemia, Temblor, Trastornos del gusto, Sequedad de boca, Reflujo, gastroesofágico, Gastroenteritis, Aumento de las transaminasas, Sequedad cutánea, Exantema exfoliativo, Rigidez musculoesquelética, Proteinuria, Aumento de la amilasa, Aumento de la lipasa, Relación internacional normalizada (INR) anómala. \| Dolor abdominal, Infección, Leucopenia, Linfopenia, Disgeusia, Mareo, Cefalea, Hipertensión, Disnea, Dolor abdominal, Estreñimiento, Ulceración de la boca, Alteración oral, Alopecia, Piel seca, Artralgia, Mialgia, Pérdida peso, Enzimas hepáticos aumentados \|  - Eventos adversos poco frecuentes   Se consideran EA poco frecuentes aquellos eventos que pueden afectar hasta 1 de cada 1.000 personas. Entre las distintas alternativas terapéuticas CCRm en líneas avanzadas se encuentran palpitaciones, arritmias, vértigo, eritema multiforme, reacción de hipersensibilidad, etc .   \| Capecitabina \| Irinotecán \| Cetuximab \| Irinotecán +Cetuximab \| Panitumumab \| Regorafenib \| FTD/TPI + BEV \| \| --- \| --- \| --- \| --- \| --- \| --- \| --- \| \| Úlceras cutáneas, rash, urticaria, reacción de fotosensibilidad, eritema palmar, hinchazón de la cara, púrpura, Obstrucción intestinal, ascitis, enteritis, gastritis, disfagia, dolor del tracto inferior abdominal, esofagitis, malestar abdominal, enfermedad de reflujo gastroesofágico, colitis, Edema, escalofríos, sintomatología gripal, rigidez, Alteración del apetito, malnutrición, diabetes, hipopotasemia, hipertrigliceridemia, Afasia, alteración de la memoria, ataxia, síncope, trastorno del equilibrio, trastornos sensoriales, neuropatía periférica, Actividad visual reducida, diplopía, embolismo pulmonar, neumotórax, hemoptisis, asma, disnea de esfuerzo, Hinchazón de las articulaciones, dolor de huesos, dolor facial, rigidez musculoesquelética, debilidad muscular, Neutropenia febril, pancitopenia, granulocitopenia, trombocitopenia, leucopenia, anemia hemolítica, Aumento del índice normalizado internacional, aumento de la creatinina sanguínea, aumento de la temperatura corporal, sangre en heces, entre otras \| En pacientes que habían sufrido episodios de deshidratación asociada a diarrea y/o vómitos, o sepsis: Insuficiencia renal, Hipotensión, Fallo circulatorio. \| Blefaritis, Queratitis, Trombosis venosa profunda, Embolia, pulmonar, Enfermedad pulmonar intersticial \| Las esperadas de ambos tratamientos. Sin evidencia de más complicaciones por combinación. \| Infección de los ojos, del párpado, Reacción anafiláctica, Queratitis ulcerosa, Queratitis, Irritación palpebral, Enfermedad pulmonar intersticial, Broncoespasmo, Sequedad nasal, Labios agrietados, secos, Necrólisis epidérmica tóxica, S. Stevens-Johnson, Necrosis cutánea, Angioedema, Hirsutismo, Uñas encarnadas, Onicolisis, Reacciones relacionadas con la perfusión. \| Reacción de Hipersensibilidad, Infarto de miocardio, Isquemia miocárdica, Crisis hipertensiva, Perforación gastrointestinal, Fístula gastrointestinal, Lesión hepática grave, Trastorno ungueal, Eritema multiforme. \| Infección tracto urinario, Gingivitis, Neutropenia febril, Pancitopenia, Hipoalbuminemia, Hiperglucemia, Neuropatía periférica, Parestesia, Disfonía, Rinorrea, Colitis, Distensión abdominal, Inflamación anal, Dispepsia, Flatulencia, Prurito, Erupción cutánea, Trastorno de las uñas, S. eritrodisestesia palmo-plantar, Pérdida de fuerza muscular, Dolor en una extremidad, Proteinuria, Pirexia, Inflamación mucosa, Dolor, Fosfatasa alcalina aumentada en sangre \|  - Afectación de los eventos adversos en la CVRS   No se dispone de datos al respecto. | | | | | | | | | | |
| Puntúe los siguientes subcriterios según considere: | | | | | | | | | | | |
| Eventos adversos graves | ☐ 5 FTD/TPI + bevacizumab es la opción más segura y tolerable | ☐ 4 | ☐ 3 | ☐ 2 | ☐ 1 | ☐ 0 | ☐ -1 | ☐ -2 | ☐ -3 | ☐ -4 | ☐ -5 FTD/TPI + bevacizumab es la opción menos segura y tolerable |
| Eventos adversos frecuentes y muy frecuentes | ☐ 5 FTD/TPI + bevacizumab es la opción más segura y tolerable | ☐ 4 | ☐ 3 | ☐ 2 | ☐ 1 | ☐ 0 | ☐ -1 | ☐ -2 | ☐ -3 | ☐ -4 | ☐ -5 FTD/TPI + bevacizumab es la opción menos segura y tolerable |
| Eventos adversos poco frecuentes | ☐ 5 FTD/TPI + bevacizumab es la opción más segura y tolerable | ☐ 4 | ☐ 3 | ☐ 2 | ☐ 1 | ☐ 0 | ☐ -1 | ☐ -2 | ☐ -3 | ☐ -4 | ☐ -5 FTD/TPI + bevacizumab es la opción menos segura y tolerable |
| Afectación de los eventos adversos en la CVRS | ☐ 5 FTD/TPI + bevacizumab es la opción más segura y tolerable | ☐ 4 | ☐ 3 | ☐ 2 | ☐ 1 | ☐ 0 | ☐ -1 | ☐ -2 | ☐ -3 | ☐ -4 | ☐ -5 FTD/TPI + bevacizumab es la opción menos segura y tolerable |
| Puntúe el criterio Seguridad en global | ☐ 5 FTD/TPI + bevacizumab es la opción más segura y tolerable | ☐ 4 | ☐ 3 | ☐ 2 | ☐ 1 | ☐ 0 | ☐ -1 | ☐ -2 | ☐ -3 | ☐ -4 | ☐ -5 FTD/TPI + bevacizumab es la opción menos segura y tolerable |
| A continuación, si lo desea puede indicar el razonamiento de su ponderación: | | | | | | | | | | | |

| CRITERIO | EVIDENCIA DISPONIBLE | | | | | | | | | | |
| --- | --- | --- | --- | --- | --- | --- | --- | --- | --- | --- | --- |
| Coste | | | | | | | | | | | |
| Subcriterios:   - Coste farmacológico - Otros costes médicos directos del tratamiento - Costes indirectos del tratamiento | - Coste farmacológico   En la siguiente tabla se muestra el coste farmacológico aproximado de las distintas alternativas terapéuticas disponibles en España para el tratamiento del CCRm en líneas avanzadas (3L+). Se presenta el coste cada 3 semanas según los PVL notificados + IVA -RDL(sanidad) disponibles en la base de datos Botplus y la pauta descrita en ficha técnica (AEMPS, 2013, 2016a, 2017, 2022) o en el EC en el caso de irinotecán + cetuximab (Cunningham et al. 2004).   \| **Tratamiento** \| **Pauta** \| **Coste/ mes*** \| \| --- \| --- \| --- \| \| FTD/TPI + BEV \| FTD/TPI: 35 mg/m^2^; 2 al día- 10 días/ 3 semanas  BEV: 5 mg/kg/ 2 semanas \| 5.434,15 € \| \| FTD/TPI \| 35 mg/m^2^; 2 al día- 10 días/ 3 semanas \| 4.104,53 € \| \| Irinotecán \| 350 mg/m2 / 3 semanas \| 179,90 € \| \| Irinotecán + Cetuximab \| Irinotecán: 350 mg/m^2^/ 3 semanas  Cetuximab: 250 mg/m^2^/ semana \| 3.103,78 € \| \| Cetuximab \| Primera dosis de 400 mg/m^2^  250 mg/m^2^/ semana el resto \| Mes 1: 3.362,47 € \| \| Mes 2 +: 2.923,88 € \| \| Panitumumab \| 6 mg/kg/ 2 semanas \| 3.193,36 € \| \| Capecitabina \| 2.500 mg/m^2^ al día- 14 días/ 3 semanas \| 83,48 € \| \| Regorafenib \| 160 mg durante 21 días / 4 semanas \| 2.308,80 € \|   Se considera un peso medio de 70 kg y una superficie corporal media de 1,72 m^2^ según las recomendaciones de la guía GENESIS (GENESIS, 2016); *la estimación mensual se realiza considerando 4 semanas  Adicionalmente, se muestra una estimación del ratio coste-efectividad incremental (RCEI) de FTD/TPI + BEV en comparación con las alternativas evaluadas. Esta estimación se ha realizado en base a la guía GENESIS(GENESIS, 2016), y considera únicamente el coste farmacológico y la eficacia en términos de SG. Esto permite determinar, en líneas generales, que FTD/TPI + BEV es una opción coste-efectiva frente al resto de alternativas (el RCEI se encuentra en todas las comparaciones por debajo del umbral de disposición a pagar establecido en 25.000€(Sacristán et al., 2020)) para el tratamiento del CCRm en líneas avanzadas. Asimismo, recientemente se ha publicado un estudio de coste-efectividad realizado por Giuliani et al. 2024(Giuliani et al., 2024) en Italia, donde se concluye que FTD/TPI + BEV puede considerarse una opción coste-efectiva para el tratamiento del CCRm en tercera línea en Italia (RCEI: 4.664,8 €).  Estos resultados junto a la evidencia mostrada en los apartados anteriores podrían demostrar que el tratamiento con FTD/TPI + BEV presenta un valor terapéutico añadido, aportando un incremento en el beneficio clínico que compensaría el coste farmacológico adicional.   \| **Tratamiento** \| **Coste/ mes** \| **Duración del Tratamiento (meses)*** \| **Coste del tratamiento** \| **SG (meses)** \| **RCEI de FTD/TPI + BEV vs.** \| \| --- \| --- \| --- \| --- \| --- \| --- \| \| FTD/TPI + BEV \| 5.434,15 € \| 5,6 \| 30.431,25 € \| 10,8 \| - \| \| FTD/TPI \| 4.104,53 € \| 2,4 \| 9.850,88 € \| 7,5 \| 6.236,48 € \| \| Irinotecan \| 179,90 € \| 7,9 \| 1.421,19 € \| 7,9 \| 10.003,47 € \| \| Irinotecan + Cetuximab \| 3.103,78 € \| 4,1 \| 12.849,65 € \| 8,6 \| 7.991,64 € \| \| Cetuximab Mes 1  Mes 2+ \| 3.362,47 € \| 3,0 \| 9.210,23 € \| 6,1 \| 4.515,11 € \| \| 2.923,88 € \| \| Panitumumab \| 3.193,36 € \| 3,0 \| 9.580,08 € \| 6,5 \| 4.849,11 € \| \| Capecitabina \| 83,48 € \| 6,1 \| 509,21 € \| 6,1 \| 6.366,39 € \| \| Regorafenib \| 3.078,40 € \| 1,9 \| 5.848,96 € \| 6,4 \| 5.586,89 € \|   RCEI: ratio coste-efectividad incremental; *En general se considera la duración del tratamiento hasta SLP, para irinotecán se considera la SG a no disponer de datos de SLP, y para cetuximab y panitumumab se considera la duración extraída del informe técnico publicado por Catsalut (CAMHDA, 2014)   - Otros costes médicos directos del tratamiento   No se dispone de datos comparativos de los costes médicos directos con cada una de las alternativas. Puesto que la opción terapéutica FTD/TPI + BEV muestra resultados de eficacia superiores al resto de opciones terapéuticas, junto con un perfil de seguridad adecuado, cabe esperar que los costes asociados a visitas médicas, visitas a urgencias, hospitalizaciones, entre otros, se reduzcan en comparación con el resto de las alternativas.   - Costes indirectos del tratamiento   Los costes indirectos se refieren al coste “social” que supone el CCRm. Es decir, la pérdida de productividad laboral entre otros, tales como pérdida de calidad de vida, interacciones sociales y/o estado emocional. Asimismo, también se consideran los costes asociados al cuidador y familiares del paciente. A pesar de que no se dispone de información sobre el impacto de las distintas alternativas sobre los costes indirectos en España, debido a los resultados de eficacia y de calidad de vida obtenidos con la opción terapéutica FTD/TPI + BEV frente a sus alternativas, cabe esperar que haya un ahorro en los costes indirectos con este fármaco. | | | | | | | | | | |
| Puntúe los siguientes subcriterios según considere: | | | | | | | | | | | |
| Coste farmacológico | ☐ 5 FTD/TPI + bevacizumab es la mejor opción | ☐ 4 | ☐ 3 | ☐ 2 | ☐ 1 | ☐ 0 | ☐ -1 | ☐ -2 | ☐ -3 | ☐ -4 | ☐ -5 FTD/TPI + bevacizumab es la peor opción |
| Otros costes médicos directos del tratamiento | ☐ 5 FTD/TPI + bevacizumab es la mejor opción | ☐ 4 | ☐ 3 | ☐ 2 | ☐ 1 | ☐ 0 | ☐ -1 | ☐ -2 | ☐ -3 | ☐ -4 | ☐ -5 FTD/TPI + bevacizumab es la peor opción |
| Costes indirectos del tratamiento | ☐ 5 FTD/TPI + bevacizumab es la mejor opción | ☐ 4 | ☐ 3 | ☐ 2 | ☐ 1 | ☐ 0 | ☐ -1 | ☐ -2 | ☐ -3 | ☐ -4 | ☐ -5 FTD/TPI + bevacizumab es la peor opción |
| Puntúe el criterio Coste en global | ☐ 5 FTD/TPI + bevacizumab es la mejor opción | ☐ 4 | ☐ 3 | ☐ 2 | ☐ 1 | ☐ 0 | ☐ -1 | ☐ -2 | ☐ -3 | ☐ -4 | ☐ -5 FTD/TPI + bevacizumab es la peor opción |
| A continuación, si lo desea puede indicar el razonamiento de su ponderación: | | | | | | | | | | | |

# **Referencias**

AEMPS. (2013). Ficha técnica de regorafenib. . Retrieved from <https://ec.europa.eu/health/documents/community-register/2016/20161014136223/anx_136223_es.pdf>

AEMPS. (2016a). Ficha técnica de trifluridine/tipiracil. . Retrieved from <https://ec.europa.eu/health/documents/community-register/2018/20180307140345/anx_140345_es.pdf>

AEMPS. (2016b). Informe de Posicionamiento Terapéutico de ramucirumab (Cyramza®) en cáncer colorrectal metastásico Retrieved from <https://www.aemps.gob.es/medicamentosUsoHumano/informesPublicos/docs/IPT-ramucirumab-Cyramza-cancer-colorrectal.pdf>

AEMPS. (2016c). Informe de Posicionamiento Terapéutico de trifluridina/tipiracil hidrocloruro (Lonsurf®) en cáncer colorrectal. Retrieved from <https://www.aemps.gob.es/medicamentosUsoHumano/informesPublicos/docs/IPT-trifluridina-Lonsurf-cancer-colorrectal.pdf>

AEMPS. (2017). Ficha técnica de Irinotecán. Retrieved from <https://cima.aemps.es/cima/dochtml/ft/70539/FichaTecnica>

AEMPS. (2022). Ficha técnica de Cetuximab. . Retrieved from <https://cima.aemps.es/cima/dochtml/ft/04281003/FT_04281003.html>

Benson, A. B., Venook, A. P., Al-Hawary, M. M., Arain, M. A., Chen, Y. J., Ciombor, K. K., . . . Gurski, L. A. (2021). Colon Cancer, Version 2.2021, NCCN Clinical Practice Guidelines in Oncology. *J Natl Compr Canc Netw, 19*(3), 329-359. doi:10.6004/jnccn.2021.0012

Botplus. (2024). Base de datos oficial del Consejo General de Colegios Oficiales de Farmacéuticos (CGCOF) Retrieved from <https://botplusweb.farmaceuticos.com/>

CAMHDA. (2014). Cetuximab, panitumumab i bevacizumab: per al tractament del càncer colorectal metastàtic en segona línia i posteriors. Barcelona, Servei Catlà de Salut.

Carrertero Colomer, M. (2005). Cáncer colorrectal metastásico. Tratamiento con un nuevo agente biológico. *Offarm, 24*, 114-116.

Cervantes, A., Adam, R., Rosello, S., Arnold, D., Normanno, N., Taieb, J., . . . clinicalguidelines@esmo.org, E. G. C. E. a. (2023). Metastatic colorectal cancer: ESMO Clinical Practice Guideline for diagnosis, treatment and follow-up. *Ann Oncol, 34*(1), 10-32. doi:10.1016/j.annonc.2022.10.003

Cervantes, A. e. a. (2023). Updated treatment recommendation for third-line treatment in advanced colorectal cancer from the ESMO Metastatic Colorectal Cancer Living Guideline. *Annals of Oncology, Volume 35, Issue 2, 241 - 243*.

Fernandez Montes, A., Alonso, V., Aranda, E., Elez, E., Garcia Alfonso, P., Gravalos, C., . . . Aparicio, J. (2023). SEOM-GEMCAD-TTD clinical guidelines for the systemic treatment of metastatic colorectal cancer (2022). *Clin Transl Oncol, 25*(9), 2718-2731. doi:10.1007/s12094-023-03199-1

Gaceta Medica. (2022). Claves en la estrategia terapéutica frente al CCRm: Medicina de Precisión e inmunoterapia. . Retrieved from <https://gacetamedica.com/investigacion/claves-estrategia-terapeutica-ccrm-medicina-precision-inmunoterapia-colorrectal-tratamiento/>

Gathirua-Mwangi, W., Yang, T., Khan, T., Wu, Y., & Afable, M. (2022). Real-world overall survival of patients receiving cetuximab in later lines of treatment for metastatic colorectal cancer. *Future Oncology, 18*(29), 3299-3310. doi:10.2217/fon-2022-0432

GENESIS, S. E. d. F. H.-G. (2016). Guía de evaluación económica e impacto prespuestario en los informes de evaluación de los medicamentos. Retrieved from <https://gruposdetrabajo.sefh.es/genesis/genesis/Documents/GUIA_EE_IP_GENESIS-SEFH_19_01_2017.pdf>

Giuliani, J., Mantoan, B., Mangiola, D., Muraro, M., Napoli, G., Tommasi, M., . . . Mandarà, M. (2024). Cost-Effectiveness of the New Combination Trifluridine/Tipiracil Plus Bevacizumab for the Third-Line Treatment for Metastatic Colorectal Cancer in Italy. *Clin Colorectal Cancer, 23*(1), 1-3. doi:10.1016/j.clcc.2023.10.005

ICO. (2020). ICO-praxis para el tratamiento medico y con irradacion de cancer colorrectal. Retrieved from <https://ico.gencat.cat/web/.content/minisite/ico/professionals/documents/arxius/ICO-ICS-Praxis-Colorrectal-2020.pdf>

Maria Ignez Braghiroli, Maria Fernanda Batistuzzo Vicentini, Leonardo Gomes da Fonseca, Karla Teixeira Souza, Renata Colombo Bonadio, Oddone Freitas Melro Braghiroli, . . . Hoff, P. M. (2022). Irinotecan combined with Panitumumab or cetuximab as third-line treatment for metastatic colorectal cancer. *Journal of Clinical Oncology, 40*.

OMS. (2022). Cáncer. Retrieved from <https://www.who.int/es/news-room/fact-sheets/detail/cancer>

Pretzsch, E., Bosch, F., Neumann, J., Ganschow, P., Bazhin, A., Guba, M., . . . Angele, M. (2019). Mechanisms of Metastasis in Colorectal Cancer and Metastatic Organotropism: Hematogenous versus Peritoneal Spread. *J Oncol, 2019*, 7407190. doi:10.1155/2019/7407190

Redecan. (2023). Estimaciones de la incidencia del cáncer en España, 2023. Retrieved from <https://redecan.org/storage/documents/02d62122-9adb-4d35-b6d0-551435dbe4ae.pdf>

Sacristán, J. A., Oliva, J., Campillo-Artero, C., Puig-Junoy, J., Pinto-Prades, J. L., Dilla, T., . . . Ortún, V. (2020). ¿Qué es una intervención sanitaria eficiente en España en 2020? *Gaceta Sanitaria, 34*, 189-193. Retrieved from <http://scielo.isciii.es/scielo.php?script=sci_arttext&pid=S0213-91112020000200015&nrm=iso>

sanidad, M. d. Actualización de deducciones de mediacamentos según el Real Decreto Ley 8/2010. Retrieved from <https://www.sanidad.gob.es/areas/farmacia/infoIndustria/infoDeducciones/ley8_2010/home.htm>

SEOM. (2024). Las cifras del cáncer en España. 2024. Retrieved from <https://seom.org/images/publicaciones/informes-seom-de-evaluacion-de-farmacos/LAS_CIFRAS_2024.pdf>

Servei Català de la Salut. (2018). Programa d’harmonització farmacoterapèutica. Cetuximab, panitumumab i bevacizumab. Per al tractament del càncer colorectal metastàtic en primera línia. Retrieved from <https://catsalut.gencat.cat/web/.content/minisite/catsalut/proveidors_professionals/medicaments_farmacia/harmonitzacio/informes/_compartits/CancerCRM-1L/informe_tecnic_CAMH_cetuximab_panitumumab_bevacizumab_CCRm_1aL.pdf>

Soler-Gonzalez, G., Sastre-Valera, J., Viana-Alonso, A., Aparicio-Urtasun, J., Garcia-Escobar, I., Gomez-Espana, M. A., . . . Girones-Sarrio, R. (2024). Update on the management of elderly patients with colorectal cancer. *Clin Transl Oncol, 26*(1), 69-84. doi:10.1007/s12094-023-03243-0

Voutsadakis, I. A. (2023). A Systematic Review and Meta-Analysis of Trifluridine/Tipiracil plus Bevacizumab for the Treatment of Metastatic Colorectal Cancer: Evidence from Real-World Series. *Curr Oncol, 30*(6), 5227-5239. doi:10.3390/curroncol30060397
