# Supplementary material for: Value contribution of trifluridine/tipiracil with bevacizumab for the treatment of metastatic colorectal cancer in Catalonia using a multicriteria decision analysis
Source: J Pharm Policy Pract. 2025 Oct 13;18(1):2567970. doi: 10.1080/20523211.2025.2567970 (PMC12710260; doi:10.1080/20523211.2025.2567970)
Supplement: Supplemental File S1 [file JPPP_A_2567970_SM8809.docx]

**Supplementary file 1 – Questionnaire 1**

Tabla de contenido

Introducción2

Criterios de ponderación3

1. Criterio 1. Epidemiología3
2. Criterio 1. Guías de práctica clínica/consensos de expertos3
3. Criterio 1. Eficacia de los tratamientos disponibles3
4. Criterio 1. Seguridad de los tratamientos disponibles4
5. Criterio 1. Coste4

Ponderación de los criterios4

1. Ponderación jerárquica de los criterios4
2. Ponderación no jerárquica de los criterios5
3. Ponderación jerárquica de los sub-criterios de epidemiología5
4. Ponderación no jerárquica de los sub-criterios de epidemiología5
5. Ponderación jerárquica de los sub-criterios de guías clínicas/consenso de expertos5
6. Ponderación no jerárquica de los sub-criterios de guías clínicas/consenso de expertos5
7. Ponderación jerárquica de los sub-criterios de eficacia6
8. Ponderación no jerárquica de los sub-criterios de eficacia6
9. Ponderación jerárquica de los sub-criterios de seguridad6
10. Ponderación no jerárquica de los sub-criterios de seguridad7
11. Ponderación jerárquica de los sub-criterios de coste7
12. Ponderación no jerárquica de los sub-criterios de coste7

Referencias8

**Introducción**

El cáncer colorrectal (CCR) es una enfermedad en la cual las células en el colon o el recto se multiplican sin control. Algunas veces se le llama simplemente cáncer de colon. El colon es una porción del intestino grueso. El recto es el canal que conecta el colon con el ano1. Algunas veces, los crecimientos de tejido anómalo, o pólipos, se forman en el colon o el recto. Con el tiempo, algunos pólipos pueden convertirse en cáncer (OMS, 2023).

De todos los cánceres, el CCR es el tercero más frecuente, con 1,1 millones de nuevos casos al año y la metástasis es la principal causa de muerte (A. Cervantes et al., 2023; Redecan, 2023). En España, el CCR es el tumor más frecuentemente diagnosticado en 2022, según datos proporcionados por la Sociedad Española de Oncología Médica (SEOM, 2024). Cabe destacar que, aproximadamente entre el 15% y el 30% de los pacientes con CCR presentan metástasis, y entre el 20% y el 50% de los pacientes con enfermedad inicialmente localizada desarrollarán metástasis. La localización más frecuente de la metástasis es el hígado, después el pulmón, el peritoneo y los ganglios linfáticos (A. Cervantes et al., 2023).

El objetivo del tratamiento sistémico es prolongar la supervivencia y mantener la calidad de vida (Fernandez Montes et al., 2023). Actualmente, las pautas de tratamiento del CCR se basan en el estadio de la enfermedad según la clasificación TNM (A. e. a. Cervantes, 2023; OMS, 2022; Servei Català de la Salut, 2018). Para la mayoría de los pacientes en estadio I y II (no se ha propagado a los ganglios linfáticos adyacentes, ni a sitios distantes) el tratamiento se basa en la cirugía, y para los pacientes en estadio III (no se ha propagado a sitios distantes) la cirugía y la quimioterapia adyuvante es el tratamiento estándar (Benson et al., 2021). En los pacientes con estadio IV (el cáncer se ha propagado a un órgano distante; es decir, hay metástasis; CCRm), el tratamiento consiste en diferentes abordajes en función de la resecabilidad del tumor primario y de las metástasis identificadas (Fernandez Montes et al., 2023; ICO, 2020). Entre las opciones farmacológicas, para el tratamiento de pacientes con CCRm estadio IV en líneas avanzadas previamente tratados con fluoropirimidinas, anti-receptor del factor de crecimiento epidérmico (EGFRs), anti-factor de crecimiento endotelial vascular (VEGF), platinos y/o irinotecanse encuentran la trifluridina-tipiracilo en monoterapia, trifluridina-tipiracilo con bevacizumab y regorafenib para pacientes (A. Cervantes et al., 2023; A. e. a. Cervantes, 2023; OMS, 2022; Servei Català de la Salut, 2018). Asimismo, existen terapias dirigidas que incluyen el retratamiento de un anti-EGFR, las cuales no tienen ningún ensayo clínico publicado que demuestre su eficacia6. Del mismo modo, según qué tipo de mutación presenta el paciente con CCRm en tercera línea se proponen otros tratamientos como inhibidores o bloqueos de las mismas mutaciones, incluso fármacos como encorafenib, ipilimumab y nivolumab en distintas combinaciones, los cuales no están financiados en España por el Sistema Nacional de Salud (SNS) para esta indicación (Sanidad, 2024; Servei Català de la Salut, 2018).

A día de hoy, uno de los pilares del continuum of care (atención continuada) del CCRm y la tendencia que hay sobre el valor de un medicamento indicado para tratar el CCRm en líneas avanzadas tras fallo de primeras líneas de tratamiento se basa en la supervivencia global (SG) o libre de progresión (SLP), así como la calidad de vida que se le ofrece al paciente con las opciones disponibles. Sin embargo, definir claramente y consensuar qué es el valor y cómo debería medirse en estos tratamientos sigue siendo un reto en la actualidad. Del mismo modo, la evaluación de medicamentos se debería fundamentar en criterios de valor que reflejen otras consideraciones más allá del precio.

Por ello, se ha propuesto obtener un consenso por parte de profesionales y decisores sobre qué es el valor y cómo debería medirse en los tratamientos indicados para el CCRm en líneas avanzadas, mediante la técnica del análisis de decisión multicriterio (MCDA, multicriteria decision analysis), la cual se puede definir como “conjunto de métodos que ayudan a las discusiones deliberativas empleando criterios explícitamente definidos” (Pericay et al., 2023).

El objetivo de este proyecto es crear un marco conceptual óptimo para la evaluación de los medicamentos en CCRm en líneas avanzadas estableciendo cuáles son los criterios que se deberían tener en cuenta para la evaluación de estos medicamentos en Cataluña, desde el punto de vista del profesional sanitario y del decisor, mediante la técnica de MCDA.

Su participación en esta fase del proyecto consiste en la ponderación jerárquica y no jerárquica de los criterios y subcriterios de valor para la evaluación de medicamentos en CCRm en líneas avanzadas en Cataluña. El objetivo de la ponderación es conocer cuales son los criterios y subcriterios más relevantes para la evaluación de medicamentos en CCRm en líneas avanzadas en Cataluña. Los resultados de la ponderación serán presentados al comité científico en la próxima reunión del 4 de abril a las 16:00h, mediante la plataforma Teams.

- La ponderación jerárquica consiste en la repartición de 100 puntos para cada criterio y posteriormente para cada subcriterio dentro de cada criterio. Los criterios y subcriterios con mayor puntuación serán considerados como los más relevantes.
- La ponderación no jerárquica consiste la asignación de una puntuación del 1 al 5 a cada uno de los criterios y subcriterios (siendo 1= nada relevante y 5= muy relevante) a cada uno de los criterios y subcriterios presentados en el cuestionario.

¿Cuál es su perfil?

- Gestor/ decisor
- Médico
- Farmacéutico hospitalario

A continuación, lea detenidamente el contenido que se presenta y responda las siguientes preguntas. Tras la ponderación de cada criterio, añada la reflexión sobre su ponderación en el espacio dedicado para ello.

Muchas gracias por su participación

# **Criterios de ponderación**

## **Criterio 1. Epidemiología**

El cáncer colorrectal (CCR) es el tercer tipo de cáncer más frecuente en el mundo. Representa aproximadamente el 10% de todos los casos de cáncer y es la segunda causa de muertes relacionadas con esta enfermedad en el mundo (OMS, 2023).

- **Incidencia y prevalencia**: Según los datos de la Red Española de Registros de Cáncer (Redecan), se estiman 42.721 casos incidentes en España para el 2023 (Redecan, 2023). No se han encontrado datos específicos de incidencia para el cáncer colorrectal metastásico (CCRm) en España, pero se conoce que aproximadamente entre el 15% y el 30% de los pacientes con CCR presentan metástasis, y entre el 20% y el 50% de los pacientes con enfermedad inicialmente localizada desarrollarán metástasis (A. Cervantes et al., 2023).

Según el documento de la Sociedad Española de Oncología Médica (SEOM) del 2024 se ha estimado que la prevalencia a los cinco años del diagnóstico de CCR a nivel mundial para el año 2020 es de 5.253.335. En España, se estimaron unos 43.370 casos para el 2022 (Fernandez Montes et al., 2023). Teniendo en cuenta que un 50% de los pacientes acaban en metástasis, correspondería a 21.685 pacientes con CCRm(Fernandez Montes et al., 2023), de los cuales el 85% pasaran a primera línea (18.432 pacientes). Para líneas avanzadas se estiman 11.981 y 4.193 pacientes en segunda y tercera línea, respectivamente (65% y 35% de los pacientes que pasan de una línea a la siguiente)(Fernandez Montes et al., 2023; Servei Català de la Salut, 2018).

- **Porcentaje respecto al resto de canceres:** Según los datos que proporciona la Organización Mundial de la Salud (OMS), el cáncer colorrectal supone casi el 20% de los 6 canceres más comunes (cáncer de mama, de pulmón, colorrectal, de próstata, de piel y gástrico) en 2020

1. **Criterio 2. Guías de práctica clínica/consensos de expertos**

Se han identificado dos guías a nivel internacional ([NCCN y ESMO) y una guía española del GEMCAD, TTD y la SEOM (Benson et al., 2021; A. e. a. Cervantes, 2023; Fernandez Montes et al., 2023). A nivel de Cataluña, se suelen seguir las guías del ICO, las ultimas guías fueron publicadas en el 2020. Asimismo, los expertos comentan que trifluridina-tipiracilo con bevacizumab debería ser evaluado por el CatSalut ya que aporta un beneficio claro. Sin embargo, trifluridina-tipiracilo en monoterapia y regorafenib se da únicamente como uso compasivo en pacientes de buen pronóstico.

Cabe mencionar, que el acceso de trifluridina-tipiracilo con bevacizumab sería más fácil ya que es un medicamento con la indicación a los pacientes con CCRm en líneas avanzadas independientemente de si tiene alguna mutación o no, es decir no sería necesario hacer la secuenciación, a diferencia de otros medicamentos que son específicos para pacientes con una mutación concreta.

- **Guías/ recomendaciones:** En líneas generales, las recomendaciones que se describen en las tres guías coinciden para los pacientes con CCRm en líneas avanzadas. Siendo la terapia de trifluridina-tipiracilo más bevacizumab (I, A) y trifluridina-tipiracilo o regorafenib (I, A) recomendada para pacientes previamente tratados con fluoropirimidinas, oxaliplatino, irinotecán y biológicos. Asimismo, cabe mencionar que la opción del retratamiento con un anti-EGFR (III,C), no tienen ningún ensayo clínico publicado que demuestre su eficacia(Fernandez Montes et al., 2023). Del mismo modo, según qué tipo de mutación presenta el paciente con CCRm en tercera línea se proponen otros tratamientos como inhibidores o bloqueos de las mismas mutaciones (III,C), incluso combinaciones de fármacos como encorafenib con cetuximab (III,C), ipilimumab con nivolumab (III,B) o cetuximab o panitumumab en monoterapia con una tomografía computarizada (II,C) (A. Cervantes et al., 2023; Fernandez Montes et al., 2023). Cabe mencionar, que algunos de estos fármacos (encorafenib, ipilimumab y nivolumab) no están financiados en España por el SNS para esta indicación (Sanidad, 2024).
- **Real World Evidence (RWE)**: Se ha considerado como subcriterio ya que hay expertos a la hora de evaluar medicamentos para CCRm en líneas avanzadas, prefieren datos de RWE a datos de ensayos clínicos. En CCRm se ha identificado un estudio a nivel nacional en el cual se menciona que en España se carece de datos reales sobre el CCRm (Pericay et al., 2023). No existen bases de datos nacionales que proporcionen información detallada sobre las características de los pacientes y de la enfermedad, las opciones de tratamiento y los resultados en oncología. Además, no se conoce la eficacia de los tratamientos sistémicos para el CCRm en la práctica clínica real en España, ni se sabe cuál es la secuencia de tratamiento óptima para maximizar la supervivencia ajustada a la calidad de vida para los diferentes grupos de pacientes (Pericay et al., 2023). Es por ello, que los expertos insisten en valorar los datos de RWE, debido a la ausencia de esto y al dato que proporciona, a diferencia de los ensayos clínicos.

1. **Criterio 3. Eficacia de los tratamientos disponibles**

El objetivo del tratamiento sistémico es prolongar la supervivencia y mantener la calidad de vida("AEMPS. 2016. Informe de Posicionamiento Terapéutico de ramucirumab (Cyramza®) en cáncer colorrectal metastásico. Consultado en enero, 2024. Disponible en: https://www.aemps.gob.es/medicamentosUsoHumano/informesPublicos/docs/IPT-ramucirumab-Cyramza-cancer-colorrectal.pdf,"). En CCRm en líneas avanzadas son especialmente importantes el estado funcional, el tipo de metástasis y la presencia de determinadas mutaciones para seleccionar el tratamiento para los pacientes (AEMPS, 2016b, 2016c)..

Se dispone de varios agentes quimioterápicos con actividad significativa en CCRm (Benson et al., 2021; A. e. a. Cervantes, 2023; Fernandez Montes et al., 2023). En primera y segunda línea se recomiendan biológicos (agentes dirigidos como anti- factor de crecimiento endotelial vascular [VEGF] y anticuerpos del receptor del factor de crecimiento epidérmico [EGFR]) en combinación con quimioterapia a la mayoría de los pacientes, a menos que estén contraindicados [I, A].

En tercera línea de CCRm (foco de este estudio), se recomienda (A. Cervantes et al., 2023; A. e. a. Cervantes, 2023):

- Trifluridina-tipiracilo más bevacizumab (European Society for Medical Oncology [ESMO]- Magnitude of Clinical Benefit Scale [MCBS]: 4) (I, A) y trifluridina-tipiracilo (ESMO- MCBS: 3) o regorafenib (ESMO- MCBS: 1) (I, A) se recomiendan para pacientes previamente tratados con fluoropirimidinas, oxaliplatino, irinotecán y biológicos.

Cabe mencionar que la opción del retratamiento con un anti-EGFR (III,C), no tienen ningún ensayo clínico publicado que demuestre su eficacia (Fernandez Montes et al., 2023). Del mismo modo, según qué tipo de mutación presenta el paciente con CCRm en tercera línea se proponen otros tratamientos como inhibidores o bloqueos de las mismas mutaciones (III,C), incluso combinaciones de fármacos como encorafenib con cetuximab (ESMO- MCBS: 4) (III,C), ipilimumab con nivolumab (ESMO- MCBS: 3) (III,B), o cetuximab o panitumumab en monoterapia con una tomografía computarizada (II,C) (A. Cervantes et al., 2023; ESMO, 2024; Fernandez Montes et al., 2023). Cabe destacar que algunos de estos fármacos (encorafenib, ipilimumab y nivolumab) no están financiados en España por el SNS para esta indicación (Sanidad, 2024).

Según las guías de práctica clínica a nivel nacional del Grupo Español Multidisciplinar de Cáncer Digestivo (GEMCAD), Grupo Español de Tumores Digestivos (TTD) y la SEOM del 2023 y a nivel internacional de la ESMO del 2022 en CCRm, y su actualización del 2023 y de la National Comprehensive Cancer Network (NCCN) del 2021 (Benson et al., 2021; A. Cervantes et al., 2023; A. e. a. Cervantes, 2023; Fernandez Montes et al., 2023), así como las publicaciones de los ensayos clínicos pivotales de las alternativas terapéuticas para el CCRm en líneas avanzadas las variables recomendadas de estudio son la SG, la SLP, la tasa de respuesta, la tasa de control de la enfermedad, duración de respuesta y tiempo transcurrido hasta el deterioro del ECOG (Grothey A, 2013; Mayer RJ, 2015).

- **Supervivencia global (SG)**: La SG se define como el tiempo desde la aleatorización hasta la muerte por cualquier causa (EMA & European Medicine Agency, 2017). Es decir, el tiempo desde la fecha de diagnóstico o el inicio del tratamiento de una enfermedad, como el cáncer, que los pacientes diagnosticados con la enfermedad siguen vivos (Institute). La SG de los tratamientos indicados para CCRm en líneas avanzadas oscilan entre 6,4 (para regorafenib) y 10,8 meses (para trifluridina-tipiracilo con bevacizumab) (Grothey A, 2013; Prager et al., 2023).
- **Supervivencia libre de progresión (SLP)**: La SLP se define como el tiempo desde la aleatorización hasta la progresión objetiva del tumor o la muerte por cualquier causa (EMA & European Medicine Agency, 2017). Es decir, el tiempo durante y después del tratamiento de una enfermedad como el cáncer, que un paciente vive con la enfermedad pero no empeora (Institute). La SLP de los tratamientos indicados para CCRm en líneas avanzadas oscilan entre 1,9 (para regorafenib) y 5,6 (para trifluridina-tipiracilo con bevacizumab) meses (Grothey A, 2013; Prager et al., 2023).
- **Tasa de respuesta**: La tasa de respuesta se define como el porcentaje de pacientes cuyo cáncer se reduce o desaparece después del tratamiento (Institute). La tasa de respuesta de los tratamientos indicados para CCRm en líneas avanzadas oscila entre el 1,0% (para regorafenib) y 6,1% (para trifluridina-tipiracilo) (Grothey A, 2013) (Clinicaltrials, 2023).
- **Tasa de control de enfermedad:** Se define como el porcentaje de pacientes con cáncer avanzado cuya intervención terapéutica ha conducido a una respuesta completa, respuesta parcial o enfermedad estable. La tasa de control de enfermedad de los tratamientos indicados para CCRm en líneas avanzadas fue alcanzada entre el 41% (para regorafenib) y el 69% (para trifluridina-tipiracilo con bevacizumab) de los pacientes (Grothey A, 2013).
- **Duración de respuesta**: Se define como el tiempo transcurrido desde la aleatorización hasta la progresión de la enfermedad o la muerte en pacientes que logran una respuesta completa o parcial (Delgado A, 2021). La duración de respuesta de los tratamientos indicados para CCRm en líneas avanzadas fue de dos meses aproxidamente para regorafenib (Grothey A, 2013).
- **Tiempo transcurrido hasta el deterioro**: Se define como el tiempo que ha transcurrido desde la aleatorización hasta el deterioro de la puntuación del estado funcional ECOG de 0 o 1 a 2 o más. En el ensayo pivotal de trifluridina-tipiracilo, se demostró que trifluridina-tipiracilo con bevacizumab demostró un retraso prolongado en el tiempo hasta el deterioro . Es importante mencionar que se entiende como deterioro, los valores establecidos en el ensayo clínico en el cual se mide esta variable. Muchas veces se utiliza el ECOG, u otras escalas que miden el estado funcional del paciente.
- **Calidad de vida relacionada con la salud (CVRS):** Los regímenes de tratamiento de primera línea más habituales en pacientes con CCRm irresecable son extremadamente tóxicas y pueden comprometer la calidad del vida del paciente, que se reconoce cada vez más como un resultado crucial en la práctica clínica y un criterio de valoración en los ensayos clínicos aleatorizados (Layos L, 2022). Debido a que uno de los objetivos del tratamiento sistémico es mantener la calidad de vida (AEMPS, 2016b), se suelen utilizar en los ensayos clínicos los cuestionarios EORTC-QLQ-C30, y EQ-5D (AEMPS, 2015, 2016b) para medirla. En ambos cuestionarios, en el caso del tratamiento de regorafenib para pacientes con CCRm en tercera línea, los resultados del ensayo pivotal sugieren que el deterioro de la calidad de vida y el estado de salud de los pacientes fue muy similar en el grupo de pacientes que recibieron regorafenib y en el grupo de pacientes que recibían placebo. Del mismo modo, en el estudio pivotal de trifluridina-tipiracilo demuestra que la calidad de vida se mantuvo en ambos brazos (trifluridina-tipiracilo en monoterapia y trifluridina-tipiracilo con bevacizumab) con una tendencia hacia un tiempo prolongado de deterioro definitivo de las escalas y subescalas de CVRS. Cabe destacar que este subcriterio es fundamental para la “humanización” de la atención al paciente. No solo se refiere al cuestionario si no a lo que el paciente reporta día a día, incluso minuto a minuto si fuera posible.

1. **Criterio 4. Seguridad de los tratamientos disponibles.**

Un evento adverso (EA) se define como un efecto adverso inesperado que se produce durante el tratamiento con un fármaco u otra terapia. Los EA pueden ser leves, moderados o graves, y pueden estar causados por algo distinto al fármaco o la terapia que se está administrando (Institute). En los ensayos clínicos, la información sobre los EA, con o sin relación causal con el fármaco o los fármacos, debe recogerse siempre y clasificarse según su gravedad (EMA & European Medicine Agency, 2017). En la seguridad se incluye el concepto tolerabilidad, definido como el grado en que los EA son aceptables para un paciente(EMA & European Medicine Agency, 2017). Cabe destacar que no solo se tiene en cuenta la gravedad del EA si no también su frecuencia. Del mismo modo, se ha de mencionar que resulta importante en seguridad conocer la vía de administración del medicamento. No es lo mismo, la administración en casa que la administración en el hospital, ya que se asegura la buena administración del medicamento por un profesional sanitario.

- **Eventos adversos graves:** Se define como cualquier acontecimiento médico adverso que, en cualquier dosis, provoque la muerte, ponga en peligro la vida, requiera la hospitalización o la prolongación de una hospitalización existente, provoque una discapacidad o incapacidad persistente o significativa, sea una anomalía congénita/defecto de nacimiento o sea un acontecimiento médicamente importante. Entre las distintas alternativas terapéuticas del CCRm en líneas avanzadas los EA graves que se pueden encontrar son: supresión de la médula ósea, toxicidad gastrointestinal, lesión hepática grave, hemorragia y perforación gastrointestinal (AEMPS, 2013, 2016a).
- **Eventos adversos muy frecuentes y frecuentes:** Se consideran EA muy frecuentes aquellos eventos que pueden afectar a más de 1 de cada 10 personas y EA frecuentes; aquellos eventos que pueden afectar hasta 1 de cada 100 personas . Entre las distintas alternativas terapéuticas del CCRm en líneas avanzadas se encuentran, infección, diarrea, astenia/fatiga, reacción cutánea mano-pie, hipertensión, disfonía, neutropenia, náuseas y leucopenia como EA muy frecuentes . Entre las distintas alternativas terapéuticas del CCRm en líneas avanzadas se encuentran enzimas hepáticos aumentadas, proteinuria, piel seca, disnea, tos, insomnio, entre otros como EA frecuentes .
- **Eventos adversos poco frecuentes**: Se consideran EA poco frecuentes aquellos eventos que pueden afectar hasta 1 de cada 1.000 personas . Entre las distintas alternativas terapéuticas CCRm en líneas avanzadas se encuentran palpitaciones, arritmias, vértigo, eritema multiforme, reacción de hipersensibilidad, etc .
- **Afectación de los EA a la CVRS**: Los EA se correlacionan significativamente con una disminución de la CVRS, independientemente del tipo de cáncer o de los tratamientos contra el cáncer utilizados (Hirose C, 2020).

1. **Criterio 5. Coste.**

El coste incluye el coste farmacológico, otros costes médicos directos del tratamiento y costes indirectos.

- **Coste farmacológico:** El coste farmacológico aproximado de las distintas alternativas terapéuticas disponibles en España para el CCRm en líneas avanzadas, durante 1 ciclo (de 28 días) ronda los 3.000 €- 4.000 € para tercera línea, según los PVL notificados + IVA -RDL disponibles en la base de datos Botplus .
- **Otros costes médicos directos del tratamiento**: estos costes se refieren a los servicios médicos como la atención hospitalaria, la atención ambulatoria, la atención primaria y la atención de urgencias, los cuales supone el 36,3%, 1,0%, 20,.% y el 7,7% en España, respectivamente (Henderson RH, 2021). Del mismo, se consideran importantes tener en cuenta el coste asociado al tratamiento y manejo de los EAs.
- **Costes indirectos del tratamiento**: Los costes indirectos se refieren al coste “social” que supone el CCRm. Es decir, la pérdida de productividad laboral entre otros, tales como pérdida de calidad de vida, interacciones sociales y/o estado emocional. Asimismo, también se consideran los costes asociados al cuidador y familiares del paciente. Sin embargo, no se ha identificado ningún estudio que cuantifique estos costes. La pérdida de productividad se produce cuando un paciente no puede trabajar con normalidad debido a la enfermedad (Farkkila et al., 2015). La pérdida de productividad se calculó multiplicando el número de días de ausencia del trabajo por el coste laboral diario medio, incluidos los pagos a la seguridad social del empleador, de una media del 38,6%, además del salario antes de impuestos (Farkkila et al., 2015). Según un estudio, realizado en Europa en el 2015 se estimó la pérdida de productividad de los pacientes con CCRm causa el 12,8% de los costes totales en el estado de enfermedad metastásica (Farkkila et al., 2015)

# **Ponderación de los criterios**

1. **Ponderación jerárquica de los criterios**

- Reparta peso sobre 100 en los siguientes criterios, en función de su relevancia para la evaluación de medicamentos en CCRm en líneas avanzadas en Cataluña. La suma total debe de ser 100

| Criterio | Ponderación |
| --- | --- |
| Epidemiología |  |
| Guias de práctica clínica/consenso de expertos |  |
| Eficacia |  |
| Seguridad |  |
| Coste |  |
| Total | 100 |

Razonamiento de su ponderación: (texto libre)

1. **Ponderación no jerárquica de los criterios**

- Puntúe del 1 al 5 los siguientes criterios (escala del 1 al 5). Siendo 1 nada relevante y 5 muy relevante para la evaluación de medicamentos en CCRm en líneas avanzadas en Cataluña. Las puntuaciones son independientes y no están relacionadas entre sí.

| Criterio | Ponderación |
| --- | --- |
| Epidemiología | 1 2  3  4  5 |
| Guias de práctica clínica/consenso de expertos | 1 2  3  4  5 |
| Eficacia | 1 2  3  4  5 |
| Seguridad | 1 2  3  4  5 |
| Coste | 1 2  3  4  5 |

Razonamiento de su ponderación: (texto libre)

# **Ponderación de los sub-criterios**

1. **Ponderación jerárquica de los sub-criterios de epidemiología**

- Reparta peso sobre 100 en los siguientes subcriterios de epidemiología, en función de su relevancia para la evaluación de medicamentos en CCRm en líneas avanzadas en Cataluña. La suma total debe de ser 100

| Subcriterio | Ponderación |
| --- | --- |
| Incidencia y prevalencia |  |
| Porcentaje respecto al resto de canceres |  |
| Total | 100 |

Razonamiento de su ponderación: (texto libre)

1. **Ponderación no-jerárquica de los sub-criterios de epidemiología**

- Puntúe del 1 al 5 los siguientes subcriterios de epidemiologia (escala del 1 al 5). Siendo 1 nada relevante y 5 muy relevante para la evaluación de medicamentos en CCRm en líneas avanzadas en Cataluña. Las puntuaciones son independientes y no están relacionadas entre sí.

| Subcriterio | Ponderación |
| --- | --- |
| Incidencia y prevalencia | 1 2  3  4  5 |
| Porcentaje respecto al resto de canceres | 1 2  3  4  5 |

Razonamiento de su ponderación: (texto libre)

1. **Ponderación jerárquica de los sub-criterios de guías de práctica clínica y consenso de expertos**

- Reparta peso sobre 100 en los siguientes subcriterios de guías de práctica clínica/consenso de expertos, en función de su relevancia para la evaluación de medicamentos en CCRm en líneas avanzadas en Cataluña. La suma total debe de ser 100

| Subcriterio | Ponderación |
| --- | --- |
| Incidencia y prevalencia |  |
| Porcentaje respecto al resto de canceres |  |
| Total | 100 |

Razonamiento de su ponderación: (texto libre)

1. **Ponderación no-jerárquica de los sub-criterios de de guías de práctica clínica y consenso de expertos**

- Puntúe del 1 al 5 los siguientes subcriterios de guías de práctica clínica/consenso de expertos (escala del 1 al 5). Siendo 1 nada relevante y 5 muy relevante para la evaluación de medicamentos en CCRm en líneas avanzadas en Cataluña. Las puntuaciones son independientes y no están relacionadas entre sí.

| Subcriterio | Ponderación |
| --- | --- |
| Incidencia y prevalencia | 1 2  3  4  5 |
| Porcentaje respecto al resto de canceres | 1 2  3  4  5 |

Razonamiento de su ponderación: (texto libre)

1. **Ponderación jerárquica de los sub-criterios de eficacia**

- Reparta peso sobre 100 en los siguientes subcriterios de eficacia, en función de su relevancia para la evaluación de medicamentos en CCRm en líneas avanzadas en Cataluña. La suma total debe de ser 100

| Subcriterio | Ponderación |
| --- | --- |
| SG |  |
| SLP |  |
| Tasa de respuesta |  |
| Tasa de control de la enfermedad |  |
| Duración de respuesta |  |
| Tiempo transcurrido hasta el deterioro |  |
| CVRS |  |
| Total | 100 |

Razonamiento de su ponderación: (texto libre)

1. **Ponderación no-jerárquica de los sub-criterios de eficacia**

- Puntúe del 1 al 5 los siguientes subcriterios de eficacia (escala del 1 al 5). Siendo 1 nada relevante y 5 muy relevante para la evaluación de medicamentos en CCRm en líneas avanzadas en Cataluña. Las puntuaciones son independientes y no están relacionadas entre sí.

| Subcriterio | Ponderación |
| --- | --- |
| SG | 1 2  3  4  5 |
| SLP | 1 2  3  4  5 |
| Tasa de respuesta | 1 2  3  4  5 |
| Tasa de control de la enfermedad | 1 2  3  4  5 |
| Duración de respuesta | 1 2  3  4  5 |
| Tiempo transcurrido hasta el deterioro | 1 2  3  4  5 |
| CVRS | 1 2  3  4  5 |

Razonamiento de su ponderación: (texto libre)

1. **Ponderación jerárquica de los sub-criterios de seguridad**

- Reparta peso sobre 100 en los siguientes subcriterios de seguridad, en función de su relevancia para la evaluación de medicamentos en CCRm en líneas avanzadas en Cataluña. La suma total debe de ser 100

| Subcriterio | Ponderación |
| --- | --- |
| EA graves |  |
| EA muy frecuentes y frecuentes |  |
| EA poco frecuentes |  |
| Afectación de los EA a la CVRS |  |
| Total | 100 |

Razonamiento de su ponderación: (texto libre)

1. **Ponderación no-jerárquica de los sub-criterios de seguridad**

- Puntúe del 1 al 5 los siguientes subcriterios de seguridad (escala del 1 al 5). Siendo 1 nada relevante y 5 muy relevante para la evaluación de medicamentos en CCRm en líneas avanzadas en Cataluña. Las puntuaciones son independientes y no están relacionadas entre sí.

| Subcriterio | Ponderación |
| --- | --- |
| EA graves | 1 2  3  4  5 |
| EA muy frecuentes y frecuentes | 1 2  3  4  5 |
| EA poco frecuentes | 1 2  3  4  5 |
| Afectación de los EA a la CVRS | 1 2  3  4  5 |

Razonamiento de su ponderación: (texto libre)

1. **Ponderación jerárquica de los sub-criterios de coste**

- Reparta peso sobre 100 en los siguientes subcriterios de coste, en función de su relevancia para la evaluación de medicamentos en CCRm en líneas avanzadas en Cataluña. La suma total debe de ser 100

| Subcriterio | Ponderación |
| --- | --- |
| Coste farmacológico |  |
| Otros costes médicos directos del tratamiento |  |
| Costes indirectos del tratamiento |  |
| Total | 100 |

Razonamiento de su ponderación: (texto libre)

1. **Ponderación no-jerárquica de los sub-criterios de coste**

- Puntúe del 1 al 5 los siguientes subcriterios de coste (escala del 1 al 5). Siendo 1 nada relevante y 5 muy relevante para la evaluación de medicamentos en CCRm en líneas avanzadas en Cataluña. Las puntuaciones son independientes y no están relacionadas entre sí.

| Subcriterio | Ponderación |
| --- | --- |
| Coste farmacológico | 1 2  3  4  5 |
| Otros costes médicos directos del tratamiento | 1 2  3  4  5 |
| Costes indirectos del tratamiento | 1 2  3  4  5 |

Razonamiento de su ponderación: (texto libre)

# **Referencias**

AEMPS. (2013). Ficha técnica de regorafenib. . Retrieved from <https://ec.europa.eu/health/documents/community-register/2016/20161014136223/anx_136223_es.pdf>

AEMPS. (2015). Informe de Posicionamiento Terapéutico de regorafenib (Stivarga®) en cáncer colorrectal. Retrieved from <https://www.aemps.gob.es/medicamentosUsoHumano/informesPublicos/docs/IPT-regorafenib-Stivarga.pdf>

AEMPS. (2016a). Ficha técnica de trifluridine/tipiracil. . Retrieved from <https://ec.europa.eu/health/documents/community-register/2018/20180307140345/anx_140345_es.pdf>

AEMPS. (2016b). Informe de Posicionamiento Terapéutico de ramucirumab (Cyramza®) en cáncer colorrectal metastásico Retrieved from <https://www.aemps.gob.es/medicamentosUsoHumano/informesPublicos/docs/IPT-ramucirumab-Cyramza-cancer-colorrectal.pdf>

AEMPS. (2016c). Informe de Posicionamiento Terapéutico de trifluridina/tipiracil hidrocloruro (Lonsurf®) en cáncer colorrectal. Retrieved from <https://www.aemps.gob.es/medicamentosUsoHumano/informesPublicos/docs/IPT-trifluridina-Lonsurf-cancer-colorrectal.pdf>

AEMPS. 2016. Informe de Posicionamiento Terapéutico de ramucirumab (Cyramza®) en cáncer colorrectal metastásico. Consultado en enero, 2024. Disponible en: <https://www.aemps.gob.es/medicamentosUsoHumano/informesPublicos/docs/IPT-ramucirumab-Cyramza-cancer-colorrectal.pdf>.

Benson, A. B., Venook, A. P., Al-Hawary, M. M., Arain, M. A., Chen, Y. J., Ciombor, K. K., . . . Gurski, L. A. (2021). Colon Cancer, Version 2.2021, NCCN Clinical Practice Guidelines in Oncology. *J Natl Compr Canc Netw, 19*(3), 329-359. doi:10.6004/jnccn.2021.0012

Botplus. (2024). Base de datos oficial del Consejo General de Colegios Oficiales de Farmacéuticos (CGCOF) Retrieved from <https://botplusweb.farmaceuticos.com/>

Cervantes, A., Adam, R., Rosello, S., Arnold, D., Normanno, N., Taieb, J., . . . clinicalguidelines@esmo.org, E. G. C. E. a. (2023). Metastatic colorectal cancer: ESMO Clinical Practice Guideline for diagnosis, treatment and follow-up. *Ann Oncol, 34*(1), 10-32. doi:10.1016/j.annonc.2022.10.003

Cervantes, A. e. a. (2023). Updated treatment recommendation for third-line treatment in advanced colorectal cancer from the ESMO Metastatic Colorectal Cancer Living Guideline. *Annals of Oncology, Volume 35, Issue 2, 241 - 243*.

Clinicaltrials. (2023). Phase III Study of Trifluridine/​Tipiracil With and Without Bevacizumab in Refractory Metastatic Colorectal Cancer Patients (SUNLIGHT). . Retrieved from <https://clinicaltrials.gov/study/NCT04737187?intr=NCT04737187&rank=1&tab=results>

Delgado A, G. A. (2021). Clinical endpoints in oncology - a primer. *Am J Cancer Res. , 15*(4), 1121-1131.

EMA, & European Medicine Agency. (2017). Guideline on the evaluation of anticancer medicinal products in man.

ESMO. (2024). ESMO-MCBS Scorecards for Nivo/Ipi (nivolumab and ipilimumab). . Retrieved from <https://www.esmo.org/guidelines/esmo-mcbs/esmo-mcbs-for-solid-tumours/esmo-mcbs-scorecards/scorecard-282-1>

Farkkila, N., Torvinen, S., Sintonen, H., Saarto, T., Jarvinen, H., Hanninen, J., . . . Roine, R. P. (2015). Costs of colorectal cancer in different states of the disease. *Acta Oncol, 54*(4), 454-462. doi:10.3109/0284186X.2014.985797

Fernandez Montes, A., Alonso, V., Aranda, E., Elez, E., Garcia Alfonso, P., Gravalos, C., . . . Aparicio, J. (2023). SEOM-GEMCAD-TTD clinical guidelines for the systemic treatment of metastatic colorectal cancer (2022). *Clin Transl Oncol, 25*(9), 2718-2731. doi:10.1007/s12094-023-03199-1

Grothey A, V. C. E., Sobrero A, Siena S, Falcone A, Ychou M, Humblet Y, Bouché O, Mineur L, Barone C, Adenis A, Tabernero J, Yoshino T, Lenz HJ, Goldberg RM, Sargent DJ, Cihon F, Cupit L, Wagner A, Laurent D;. (2013). CORRECT Study Group. Regorafenib monotherapy for previously treated metastatic colorectal cancer (CORRECT): an international, multicentre, randomised, placebo-controlled, phase 3 trial. *Lancet. , 26*(9863), 303-312. doi:10.1016/S0140-6736(12)61900-X

Henderson RH, F. D., Maughan T, Adams R, Allemani C, Minicozzi P, Coleman MP, McFerran E, Sullivan R, Lawler M. . (2021). The economic burden of colorectal cancer across Europe: a population-based cost-of-illness study. *Lancet Gastroenterol Hepatol., 6*, 709-722. doi:10.1016/S2468-1253(21)00147-3

Hirose C, F. H., Iihara H, Ishihara M, Nawa-Nishigaki M, Kato-Hayashi H, Ohata K, Sekiya K, Kitahora M, Matsuhashi N, Takahashi T, Okuda K, Naruse M, Ishihara T, Sugiyama T, Yoshida K, Suzuki A.,. (2020). Real-world data of the association between quality of life using the EuroQol 5 Dimension 5 Level utility value and adverse events for outpatient cancer chemotherapy. *Support Care Cancer., 28*, 5943-5952. doi:10.1007/s00520-020-05443-8

ICO. (2020). ICO-praxis para el tratamiento medico y con irradacion de cancer colorrectal. Retrieved from <https://ico.gencat.cat/web/.content/minisite/ico/professionals/documents/arxius/ICO-ICS-Praxis-Colorrectal-2020.pdf>

Institute, N. C. Dictionary of Cancer Terms. Retrieved from <https://www.cancer.gov/publications/dictionaries/cancer-terms/def/overall-survival>

Layos L, M.-B. E., Ruiz de Porras V. Curcumin. (2022). A Novel Way to Improve Quality of Life for Colorectal Cancer Patients? *Int J Mol Sci., 14*(22), 1405. doi:10.3390/ijms232214058

Mayer RJ, V. C. E., Falcone A, Yoshino T, Garcia-Carbonero R, Mizunuma N, Yamazaki K, Shimada Y, Tabernero J, Komatsu Y, Sobrero A, Boucher E, Peeters M, Tran B, Lenz HJ, Zaniboni A, Hochster H, Cleary JM, Prenen H, Benedetti F, Mizuguchi H, Makris L, Ito M, Ohtsu A;. (2015). RECOURSE Study Group. Randomized trial of TAS-102 for refractory metastatic colorectal cancer. *N Engl J Med, 14*(20), 1909-1919. doi:10.1056/NEJMoa1414325

OMS. (2022). Cáncer. Retrieved from <https://www.who.int/es/news-room/fact-sheets/detail/cancer>

OMS. (2023). Cáncer colorrectal. . Retrieved from <https://www.who.int/es/news-room/fact-sheets/detail/colorectal-cancer>

Pericay, C., Fernandez Montes, A., Alonso Orduna, V., Macias Declara, I., Asensio Martinez, E., Rodriguez Salas, N., . . . Cirera, L. (2023). Real-World Outcomes in Patients with Metastatic Colorectal Cancer in Spain: The RWD-ACROSS Study. *Cancers (Basel), 15*(18). doi:10.3390/cancers15184603

Prager G, T. J., Fakih M, et al. . (2023). O-9 health-related quality of life associated with trifluridine/tipiracil in combination with bevacizumab in refractory metastatic colorectal cancer: An analysis of the phase 3 sunlight trial. . *Annals of Oncology., 34*. Retrieved from <https://www.annalsofoncology.org/action/showPdf?pii=S0923-7534%2823%2900166-7>

Prager, G. W., Taieb, J., Fakih, M., Ciardiello, F., Van Cutsem, E., Elez, E., . . . Investigators, S. (2023). Trifluridine-Tipiracil and Bevacizumab in Refractory Metastatic Colorectal Cancer. *N Engl J Med, 388*(18), 1657-1667. doi:10.1056/NEJMoa2214963

Redecan. (2023). Estimaciones de la incidencia del cáncer en España, 2023. Retrieved from <https://redecan.org/storage/documents/02d62122-9adb-4d35-b6d0-551435dbe4ae.pdf>

Sanidad, M. d. (2024). BIFIMED: Buscador de la Información sobre la situación de financiación de los medicamentos - Nomenclátor de FEBRERO - 2024. Retrieved from <https://www.sanidad.gob.es/profesionales/medicamentos.do>

SEOM. (2024). Las cifras del cáncer en España. 2024. Retrieved from <https://seom.org/images/publicaciones/informes-seom-de-evaluacion-de-farmacos/LAS_CIFRAS_2024.pdf>

Servei Català de la Salut. (2018). Programa d’harmonització farmacoterapèutica. Cetuximab, panitumumab i bevacizumab. Per al tractament del càncer colorectal metastàtic en primera línia. Retrieved from <https://catsalut.gencat.cat/web/.content/minisite/catsalut/proveidors_professionals/medicaments_farmacia/harmonitzacio/informes/_compartits/CancerCRM-1L/informe_tecnic_CAMH_cetuximab_panitumumab_bevacizumab_CCRm_1aL.pdf>
